# Engineering Betalain Biosynthesis in Tomato for High Level Betanin Production in Fruits

**DOI:** 10.3389/fpls.2021.682443

**Published:** 2021-06-09

**Authors:** Ramona Grützner, Ramona Schubert, Claudia Horn, Changqing Yang, Thomas Vogt, Sylvestre Marillonnet

**Affiliations:** Department of Cell and Metabolic Biology, Leibniz Institute of Plant Biochemistry, Halle, Germany

**Keywords:** betalains, betanin, cyclo-DOPA, metabolic pathway engineering, plant secondary metabolites, heterologous host, food colorant

## Abstract

Betalains are pigments found in plants of the Caryophyllales order, and include the red-purple betacyanins and the yellow-orange betaxanthins. The red pigment from red beets, betanin, is made from tyrosine by a biosynthetic pathway that consists of a cytochrome P450, a L-DOPA dioxygenase, and a glucosyltransferase. The entire pathway was recently reconstituted in plants that do not make betalains naturally including potato and tomato plants. The amount of betanin produced in these plants was however not as high as in red beets. It was recently shown that a plastidic arogenate dehydrogenase gene involved in biosynthesis of tyrosine in plants is duplicated in *Beta vulgaris* and other betalain-producing plants, and that one of the two encoded enzymes, BvADHα, has relaxed feedback inhibition by tyrosine, contributing to the high amount of betanin found in red beets. We have reconstituted the complete betanin biosynthetic pathway in tomato plants with or without a *BvADHα* gene, and with all genes expressed under control of a fruit-specific promoter. The plants obtained with a construct containing *BvADHα* produced betanin at a higher level than plants obtained with a construct lacking this gene. These results show that use of BvADHα can be useful for high level production of betalains in heterologous hosts. Unlike red beets that produce both betacyanins and betaxanthins, the transformed tomatoes produced betacyanins only, conferring a bright purple-fuschia color to the tomato juice.

## Introduction

The large diversity of plants, fungi and microorganisms found in various habitats around the planet produce a vast array of secondary metabolites. These compounds are made to increase the fitness of plants to the environment, and serve to attract beneficial organisms such as pollinators or seed dispersers, or to protect them against pathogens, predators, herbivores or environmental stress. Many of these metabolites can also have beneficial properties for human use, and can be used, for example, as flavor compounds, natural food colorants, health promoting compounds or medicinal ingredients. These beneficial molecules are, however, not always produced in the natural hosts in high amount. In addition, the host organisms may not always be well suited to grow in a standard agricultural or industrial setting. Therefore, transferring the entire biosynthetic pathway that synthesizes these metabolites into organisms that can be grown in standard industrial or agricultural settings provides a solution for producing these natural compounds in large amount economically (Staniek et al., 2013; Owen et al., 2017).

Host organisms suitable for production of beneficial metabolites include microorganisms such as *E. coli* or yeasts, or multicellular organisms such as plants. In plants, compounds of interest may be produced in vegetative tissues such as leaves, in fruits or in storage organs such as roots or tubers. In any case, all genes of the pathway need to be cloned under control of promoters that will direct expression in the desired tissue and at the right time. This requires having access to several promoters that are active not only at the same time and in the same tissue, but also in the same cell type, for all genes of the pathway. Unfortunately, a library of promoters with these characteristics is rarely available. One simple solution to this problem consists of cloning all genes of the pathway under control of the same promoter, which would ensure perfect co-expression of these genes. Such constructs will, however, contain repeats and may be unstable in either *E. coli*, Agrobacterium, or in plants after transformation. Moreover, this solution does not allow control of the relative level of expression of the various genes of the pathway. An alternative strategy consists of fusing the coding sequences of all genes of the pathway with the 2A peptide (He et al., 2020). This strategy facilitates the preparation of constructs, as there is only one coding sequence, and there is need for only one promoter and terminator. It is, however, limited by the fact that fusion of several genes may lead to lower expression of the genes, especially when many genes are required. In addition, this strategy does not allow control of the relative level of expression of the various genes of the pathway.

A different strategy provides a solution to these limitations. It consists of cloning all genes of the pathway under control of natural or synthetic promoters that are responsive to a transcriptional activator. The transcription factor can be cloned in the same construct and expressed under control of a tissue-specific promoter, and can come from the same plant or from heterologous organisms such as yeast or bacteria (Bruckner et al., 2015; Liu and Stewart, 2016; Li et al., 2017; Selma et al., 2019; Belcher et al., 2020; Cai et al., 2020). An example of a transcriptional activator of bacterial origin consists of transcription activator-like (TAL) effectors. TAL effectors are virulence factors from bacterial plant pathogens such as *Xanthomonas campestris*. TAL effectors are secreted into plant cells, where they reach the nucleus and bind the promoter region of target genes, resulting in transcriptional activation of these genes (Kay et al., 2007). TAL effectors can be reprogrammed to bind any DNA sequence of choice by swapping repeats from the repeat region of TAL effectors (Boch et al., 2009). Libraries of promoters made from degenerate sequences that flank an 18 bp TAL effector binding sequence were shown to be able to drive a range of expression levels when bound to a given dTALE (Bruckner et al., 2015). These synthetic TAL activated promoter (STAP) libraries have the benefit of a relatively small size (about 100 bp), which is convenient for cloning of multigene constructs. This strategy of using transcription factors and associated promoter libraries therefore provides the advantage of allowing a choice of promoters to provide a desired level of expression for each gene of the pathway.

In this manuscript we have tested the strategies that rely on the reuse of the same promoter multiple times, or on the use of a transcription factor. As an example of a metabolic pathway, we have chosen to reconstitute the betalain biosynthetic pathway in tomato. Betalains are tyrosine-derived compounds that are made in some plants of the caryophyllales order, which includes cacti, bougainvillea bushes, and beets such as beetroot. Betalains include the red-purple betacyanins and the yellow-orange betaxanthins and can be used as food colorants (Esatbeyoglu et al., 2015). Betalains are also known to have powerful antioxidant properties and their consumption is thought to provide many benefits for human health (Gandia-Herrero et al., 2016). Despite these beneficial properties, betalains are not frequently consumed as they are not present in many fruits or vegetables. It would therefore be beneficial to produce betalains in vegetable or fruits that are more commonly consumed. Transgenic plants expressing a betanin biosynthetic pathway have recently been produced for a range of species including egg plants, potatoes and tomato (Polturak et al., 2016, 2017). Unfortunately, the tomato plants that were obtained so far produced betanin in the fruit at a relatively low level. In plants, the precursor of betalains, tyrosine, is made from prephenate by a prephenate dehydrogenase, and from arogenate by an arogenate dehydrogenase (ADH). Enzymes encoded by these genes are normally strongly inhibited by tyrosine (Lopez-Nieves et al., 2018). In *Beta vulgaris* (red beet), two ADH enzymes are encoded by two duplicated genes (*BvADHα* and *BvADHβ*) (Lopez-Nieves et al., 2018). Interestingly, BvADHα has relaxed sensitivity to tyrosine, contributing to the high level of betalain biosynthesis in *B. vulgaris*, and especially in red beet cultivars. The same gene duplication is also present in other plants of the Caryophyllales order that make betalains. It was recently shown that transient expression of *ADHα* and of the betanin biosynthesis genes in *Nicotiana benthamiana* leaves led to a dramatic increase in betalain biosynthesis, relative to infiltrations where ADHβ was used (Timoneda et al., 2018).

In this work, we have made transgenic tomato plants expressing all genes necessary for betanin biosynthesis, and an *ADHα* gene to ensure sufficient availability of the tyrosine precursor. Several construct types were tested, including constructs containing the same promoter used multiple times or constructs that rely on a TAL effector transcription factor for transcriptional activation of all genes of the pathway. While TAL effector constructs successfully co-expressed all genes in transient assays, they could not be used for tomato transformation, but should be suitable for use in other species. Constructs relying the same promoter for all genes of the pathway led to production of transgenic plants that produced betanin in a very high amount.

## Materials and Methods

### Plant Material

Transient infiltration experiments were made in a standard *N. benthamiana* line. *Solanum lycopersicum* line Micro Tom was used for plant transformation. The transformants were backcrossed in the determinant tomato cultivar M82 and the indeterminant cultivar Moneymaker. Fresh beetroots purchased from a supermarket were used for pigment extraction for LC/MS analyses and for spectrometry analysis for betanin quantification.

### Generation of DNA Constructs

Constructs were made using the modular cloning system MoClo (Engler et al., 2014; Marillonnet and Grutzner, 2020). Coding sequences of the betanin biosynthesis genes BvCYP76AD1 and BvDODA1 were amplified by PCR from red beet cDNA and cloned in the level 0 cloning vector pICH41308 (constructs pAGM7535 and pAGM7523, respectively). The betanidin glucosyltransferase, Db5GT, was amplified from *Dorotheanthus bellidiformis* cDNA (level 0 construct pAGM7547). The coding sequence of the arogenase dehydrogenase BvADHα was amplified by PCR from red beet cDNA and cloned as level 0 module (construct pAGM55351). Sequence of the four level 0 modules is given in Supplementary Figure 1. The coding sequences were then subcloned in level 1 cloning vectors for transient expression in *N. benthamiana.* The level 1 cloning vectors for three of the genes contain a Bar expression cassette (used as buffer sequence) located between the terminator of the transcription unit and the right border. This is because we have observed that transcription units with short terminators located close to the right border sometimes have a low expression level. The tobacco mosaic virus 3′NTR was cloned between the BvDODA1 coding sequence and the Ocs terminator to increase expression of this gene, as viral NTR sequences are known to increase expression of the upstream coding sequences. The list of parts and modules for making the two constructs for tomato transformation, pAGM48488 and pAGM51753, are shown in Supplementary Figure 2. The level 0 parts, the level 1 expression constructs, as well as a level 1 cloning vector for transient expression in *N. benthamiana* have been deposited to Addgene (list of parts and constructs shown in Supplementary Figure 3).

### Transient Expression in *N. benthamiana*

Constructs were transformed in Agrobacterium strain GV3101:pMP90. The transformed Agrobacterium strains were grown at 28°C in LB medium supplemented with rifampicin and either carbenicillin for level 1 constructs, or kanamycin for level 2 constructs (all at 50 μg/ml). The cultures were diluted to an OD600 of 0.2 in infiltration solution containing 10 mM MES pH 5.5 and 10 mM MgSO_4_ and were infiltrated in leaves of greenhouse-grown *N. benthamiana* plants using a syringe without a needle.

### Plant Transformation and Transgenic Plants Cultivation

Tomato stable transformation was performed by agrobacterium inoculation of cotyledons from 8 day old seedlings as described in Schubert et al. (2019). In short, pre-conditioned cotyledon pieces were incubated for 30 min in an agrobacterium suspension with an OD600 of 0.08, transferred to co-cultivation media and kept in darkness. After 2 days, cotyledon pieces were placed on selection media under long-day conditions. Every 2 weeks, plantlets were transferred to fresh media with decreasing cytokinin (trans-Zeatinriboside) und constant auxin (IAA) concentration and final substitution by gibberellic acid. Finally, plantlets were cultivated on phytohormone-free rooting media until root development occurred and transferred into soil. Transgenic plants were grown in the greenhouse under long-day conditions with the following conditions: day, 26°C, night 23°C, 70% humidity.

### LC-MS Analysis of Betanin and Related Metabolites

For all samples, 12 mg of tissue were harvested in 2 ml tubes and frozen in liquid nitrogen. Three steel beads were added per tube, and the samples were ground two times for 30 s at 30 Hz with an electric mill. One hundred and twenty microliter of methanol buffer (50% methanol, 1 mM ascorbic acid, 0.5% formic acid) was added, and the samples were vortexed and then incubated on ice for 15 min. The samples were spun at 13,000 rpm for 10 min at 4°C. The supernatant was centrifuged one more time. Two to ten microliter of the supernatant was used for the HPLC analysis.

*N. benthamiana* leaf or tomato fruit extracts were analyzed by reversed-phase HPLC/ESI-MS on a Nucleoshell RP_18_ 100/4 mm column (Machery-Nagel, Düren, Germany) at a flow rate of 0.6 ml/min with a gradient of 2% solvent B (acetonitrile) up to 22% B in solvent A (0.1% formic acid) at a rate of 1%/min within 20 min using an Alliance e2695 chromatography system (Waters, Eschborn, Germany), equipped with a Waters 2996 photodiode array and a Waters QDA detector, respectively. For separation of cyclo-DOPA-5-*O*-glucoside, after derivatization with 4-dimethylamino cinnamaldehyde (DMACA), a comparable gradient, up to 32% solvent B in A within 20 min was used. Compounds were identified by UV/VIS in maxplot detection from 240 to 600 nm and ESI-MS between m/z 300 and 800 in positive ionization mode, cone voltage set at 15 V, and analyzed using the Empower 3 software (Waters). A standard of betanin (betanidin-5-*O*-β-D-glucopyranoside) was obtained from Merck (Darmstadt, Germany). Its positional isomer gomphrenin I (betanidin-6-*O*-β-D-glucopyranoside) was previously isolated from flowers of *Gomphrena globosa* (Heuer et al., 1992). Identification of betacyanins was based on a combination of retention times, spectral properties, mass signals, and standards of betanin and gomphrenin I. Betaxanthins were identified by UV-spectra and molecular masses based on reference data from the literature.

### Spectrophotometric Quantification

Betanin quantification was performed in triplicate for each sample. Therefore, for each sample, three aliquots of frozen tissue of between 20 and 50 mg were weighted and added to three 2 ml tubes. Extraction was performed in the same manner as the samples for LC/MS analysis, with a volume of methanol buffer added corresponding to the weight of tissue (for example 360 μL of buffer for 36 mg of tissue). For quantification, the samples were diluted 12-fold with double distilled water. The diluted samples were measured using the method described by Stintzing et al. (2003). The betacyanin (betanin and isobetanin) content (BC) was calculated using the equation BC [mg/l] = [(A × DF × MW × 1,000)/(*varepsilon* × L)], where A is the absorption at 538 nm (for betacyanins), DF is the dilution factor, L is the path length of the 1 cm cuvette. For betanin, MW = 550 g/mol, and the molar extinction coefficient ε = 60,000 l/mol cm in H_2_O. All extracts were measured by the absorption at 538 nm to quantify the betacyanin content (betanin and isobetanin).

### Identification of the T-DNA Insertion Site Sequence

For identification of flanking T-DNA sequences, genomic DNA of the transformant was extracted using the Nucleospin Plant II Kit from Macherey Nagel. DNA was G-tailed using dGTP and terminal transferase (NEB cat M0315S) using the recommended protocol (37°C incubation for 30 min followed by 10 min at 70°C). G-tailing takes place at random DNA breaks that occur during DNA extraction. A first PCR was made with the G-Tail specific primer bap2pc (gtccagagccgtccagcaac ccccccccccccc) and a T-DNA specific primer amin3 (gttccctctgctgatatggctgag) or amin5 (gcgcgcaaactaggataaattatcgcg) for T-DNA sequences near the right border, or nosan1 (cgggggtcataacgtgactcc) for T-DNA sequences near the left border. A second nested PCR was performed using primers bap2 (gtccagagccgtccagcaac) and amin5 (nested for amin3, gcgcgcaaactaggataaattatcgcg), amin6 (nested for amin5, cgcggtgtcatctatgttactagatcg) or nosan2 (nested for nosan1, ttctccgctctccggatccgaa). The PCR products (often a smear, as G-tailing occurs at random positions flanking the T-DNA) were cloned in vectors containing homologous sequences with the ends of the PCR products (homology to the Bap2pc primers and with the end of the T-DNA) using a homology directed cloning protocol, Quick and Clean cloning (Thieme et al., 2011). Inserts in the clones obtained were then sequenced with vector-specific primers.

### Preparation of Colored Lemonade and Yogurt

For red beets and fruits from the tomato cross M82 × pAGM51753 pt2, stock solutions were prepared by adding 8.6 g of ground tissue, 50 g of sugar and water to a final volume of 100 ml. The solution was filtered through a fine sieve to remove ground flesh tissues. The stock solution was heated until dissolution of the sugar. For blackcurrant, a bottle of pure juice was purchased in a supermarket. Since it did not contain flesh, a stock solution was made from 4.3 g of juice with 50 g of sugar to a final volume of 100 ml. Lemonade was made by adding 6 ml of stock solution, 3 ml of lemon juice and water to a final volume of 100 ml. A diluted lemonade mix was made by adding 2 ml of stock solution, 4 ml of sugar solution (50 g/100 ml), 3 ml of lemon juice and water for a final volume of 100 ml. For preparation of colored yogurt, 3 g of juice directly squeezed from beetroot or tomato fruits using a garlic press was added to 30 g of yogurt.

## Results

### Betanin Production by Transient Expression in *Nicotiana benthamiana*

**FIGURE 1:**
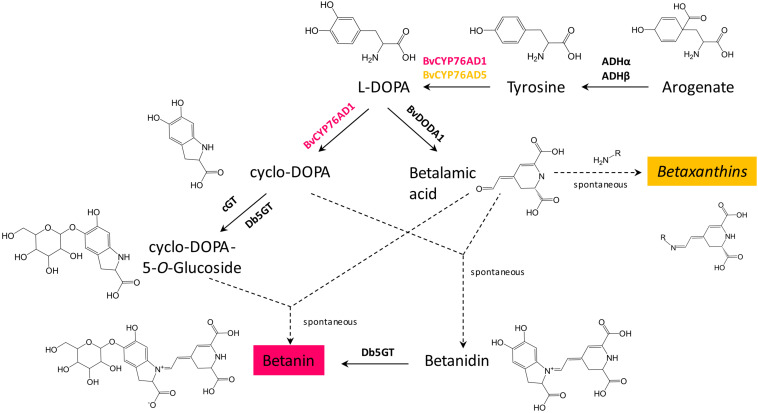
Genes involved in betalain biosynthesis. Two genes are involved in biosynthesis of L-DOPA from tyrosine, BvCYP76AD1 (shown in red, as it is mostly involved in betacyanins biosynthesis) and BvCYP76AD5 (shown in yellow, as it is involved in biosynthesis of betaxanthins). In *Beta vulgaris*, two arogenate dehydrogenases, ADHα and ADHβ, are involved in biosynthesis of the precursor of betalains, tyrosine. Dashed arrows indicate spontaneous reactions.

**FIGURE 2:**
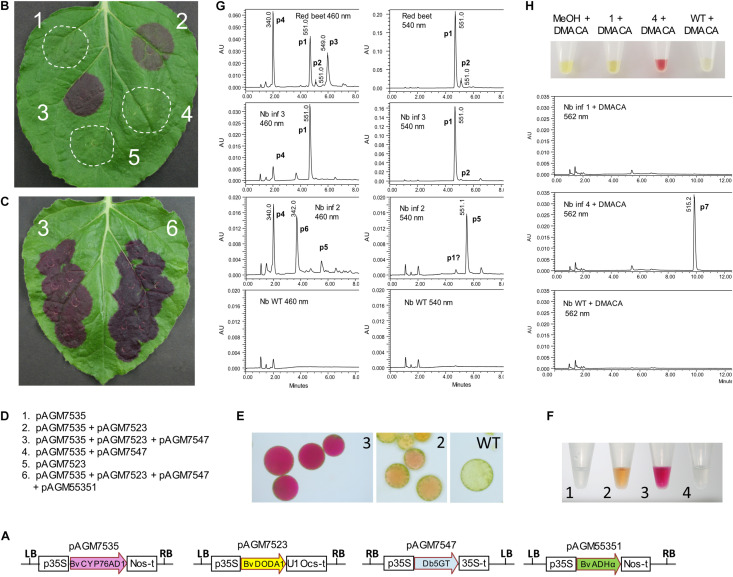
Betalain biosynthesis by transient expression in *Nicotiana benthamiana* leaves. **(A)** Structure of the level 1 constructs for transient expression of the genes involved in betalain biosynthesis. p35S, 35S promoter; Nos-t, Nos terminator; U1 Ocs-t, 3′ untranslated sequences from TMV U1 viral genome followed by Ocs terminator; 35S-t, 35S terminator; LB and RB, left and right T-DNA borders. **(B,C)** Transient expression of the level 1 constructs in *N. benthamiana* leaves. Pictures taken 5 days after infiltration. **(D)** Description of the combination of constructs infiltrated in the leaves shown in **(B,C)**. **(E)** Protoplasts prepared from infiltrations 2 and 3 from **(B)** and from WT non-infiltrated tissue. **(F)** Extracts from infiltrations 1 to 4 from **(B)**. **(G)** LC/MS analysis of extracts 2 and 3 shown in **(F)** and of a beetroot sample as a control. Note that the scale range (AU) is not the same for all samples. Identified peaks with the same retention time across runs: p1, betanin; p2, isobetanin, p3, neobetanin; p4, vulgaxanthin I; p5, gomphrenin; p6, unidentified peak with a UV spectrum characteristic of betaxanthins. **(H)** The addition of 4-dimethylaminocinnamaldehyde (DMACA) to extracts 1 and 4 from **(F)** and from a WT extract gives a red color for extract 4 due to a spontaneous Schiff base formation with cyclo-DOPA-glucoside, produced upon infiltration. LC/MS analysis shows the corresponding reaction product at λmax 562 nm (peak 7).

Three genes are required for betanin biosynthesis: a cytochrome P450 to make L-DOPA and cyclo-DOPA from tyrosine (BvCYP76Ad1 in *B. vulgaris*), an L-DOPA dioxygenase to make betalamic acid from L-DOPA (BvDODA1 in *B. vulgaris*), and a glucosyltransferase to convert betanidin to betanin (Db5GT in *Dorotheanthus bellidiformis*, Figure 1; Vogt et al., 1999; Hatlestad et al., 2012). Another gene, a cyclo-DOPA glucosyltransferase (cGT) can also be involved in betanin biosynthesis by converting cyclo-DOPA to cyclo-DOPA-5-*O*-glucoside, which then spontaneously reacts with betalamic acid to produce betanin (Sasaki et al., 2004, 2005). Coding sequences of the first two genes, BvCYP76AD1 and BvDODA1, were amplified by PCR from red beet cDNA. The glucosyltransferase (5GT) was amplified from *D. bellidiformis* cDNA (Db5GT). This glucosyltransferase was the first enzyme of the betalain biosynthesis pathway that was identified (Vogt et al., 1999), but it had not yet been used to reconstitute a betalain biosynthesis pathway in heterologous hosts. To check that all three genes were functional for betanin biosynthesis, they were subcloned in binary vectors under the control of the 35S promoter for expression in plants (Figure 2A). Different combination of genes genes were then transiently expressed in *N. benthamiana* leaves by infiltration of Agrobacterium suspensions (Figure 2D).

Co-expression of the three genes led to dark purple color in the infiltrated leaf area, starting 2 days after infiltration, and gaining in intensity the following days (Figure 2B, infiltration 3). Protoplasts of the infiltrated leaf area also revealed a strong fuchsia color in the vacuole (Figure 2E). An extract of the infiltrated leaf area revealed intense purple color, as expected for the presence of betanin (Figure 2F). This extract was analyzed by LC/MS and compared to an extract from red beet. Analysis of the red beet extract revealed major peaks for betanin (peak 1, or m/z 551.1 [M+H]^+^, λ_max_ = 538 nm Figure 2G), and isobetanin (peak 2, m/z 551.1 [M+H]^+^, λ_max_ = 538 nm), a peak of neobetanin presumably (peak 3, m/z 549.0 [M+H]^+^, λ_max_ = 469 nm) and a peak of glutamine betaxanthin (vulgaxanthin I) (peak 4, m/z 340.1 [M+H]^+^, λ_max_ = 468 nm), both described previously from red beet (Alard et al., 1985). Analysis of the extract from the leaf sector expressing all three betanin biosynthesis genes (infiltration 3) revealed a strong peak of betanin (peak 1, Figure 2G), which was, for most samples, the only peak. In some samples, a very small peak of vulgaxanthin I that overlapped with a background peak found in uninfiltrated *N. benthamiana* leaf. The absence or only minute amounts of vulgaxanthin I confirms published results indicating that a different cytochrome P450 (CYP76AD5) is required for efficient biosynthesis of betaxanthins (Polturak et al., 2016; Sunnadeniya et al., 2016).

Co-expression of BvCYP76AD1 and BvDODA1 without a glucosyltransferase is expected to lead to the production of betanidin. Purple-brown color was produced in the leaf area where these two genes were co-expressed (Figure 2A, infiltration 2). An extract made from the infiltrated leaf area resulted in yellow-orange color (Figure 2F). LC/MS analysis of this extract did not show the presence of betanidin, but revealed the presence of a metabolite with the same mass as betanin (peak 5, m/z 551.1 [M+H]^+^, λ_max_ = 538 nm, Figure 2G), but with a retention time identical to that from a gomphrenin I standard (Supplementary Figure 4), the positional isomer of betanin. In addition, a small amount of vulgaxanthin I (peak 4) and an unidentified peak with a mass of m/z 342.1 [M+H]^+^, λ_max_ = 538 nm (peak 6) with a UV spectrum characteristic of betaxanthins could be detected. The relative amount of these compounds varied in different leaves harvested at different time after infiltration (Supplementary Figure 5) but was on average in lower quantity than the amount of betanin made in infiltrations with a complete betanin biosynthetic pathway. The aglycone, betanidin was not detected. It was most likely produced, and, as it is known to be unstable, was either degraded or converted to gomphrenin I by the presumably promiscuous specificity of a *N. benthamiana* glycosyltransferase. A very small peak of the same mass and retention time as betanin could also be detected (peak p1). This peak was in fact an overlay of several peaks, and may contain traces of betanin synthesized by a second non-specific flavonoid glucosyltransferase *N. benthamiana* enzyme.

BvCYP76AD1 is the first enzyme of the betanin biosynthesis pathway, and is required for conversion of tyrosine to L-DOPA. Therefore, as expected, infiltration of BvDODA1 alone or in combination with the glucosyltransferase did not lead to any visible sign of betalain biosynthesis (Figures 2A,B). No color was detected in the leaf extract.

Infiltration of the Cyp76AD1 and Db5GT did not result in any color in the infiltrated leaf. If Db5GT works as a cyclo-DOPA glucosyltransferase in addition to the previously reported betanidin 5-glucosyltransferase activity, this infiltration should lead to formation of cyclo-DOPA-5-O-glucoside. To detect the potential presence of this compound, cyclo-DOPA-5-O-glucoside was derivatized by adding 4-dimethylaminocinnamaldehyde (DMACA) to an extract from the infiltrated area. The color of the extract immediately turned purple (Figure 2H), indicative of a Schiff base formation between the aldehyde and the amino group of cyclo-DOPA-5-*O*-glucoside, resulting in an aldimine with similar spectral properties as betanin. LC/MS analysis of the reaction product revealed the expected peak with a mass of m/z 515.2 [M+H]^+^ and a λ_max_ of 561 nm, indicating that cyclo-DOPA-5-*O*-glucoside is indeed made. This proves that the Db5GT has cyclo-DOPA GT activity in addition to the previously reported betanidin 5-GT activity.

### ADHα Enhances Betanin Production in *N. benthamiana*

The coding sequence of BvADHα was amplified by PCR from red beet cDNA and subcloned in an expression construct under control of the 35S promoter (pAGM55351). To check that this construct was functional, we co-expressed it with the genes for betalain biosynthesis described above (pAGM7535/BvCYP76AD1, pAGM7523/BvDODA1, and pAGM7547/Bv5GT). These constructs were transiently expressed in *N. benthamiana* leaves, with or without the ADHα construct (Figure 2C). A few days after infiltration, it was possible to see that more betanin was produced when the ADHα was present. The amount of betanin was quantified by spectrophotometry. The amount of betanin produced in *N. benthamiana* increased from 700 mg/kg of fresh weight without ADHα to 2.5 g/kg of fresh weight with ADHα (Supplementary Figure 6).

### Expression of the Betalain Pathway Under Control of a TAL Effector

**FIGURE 3:**
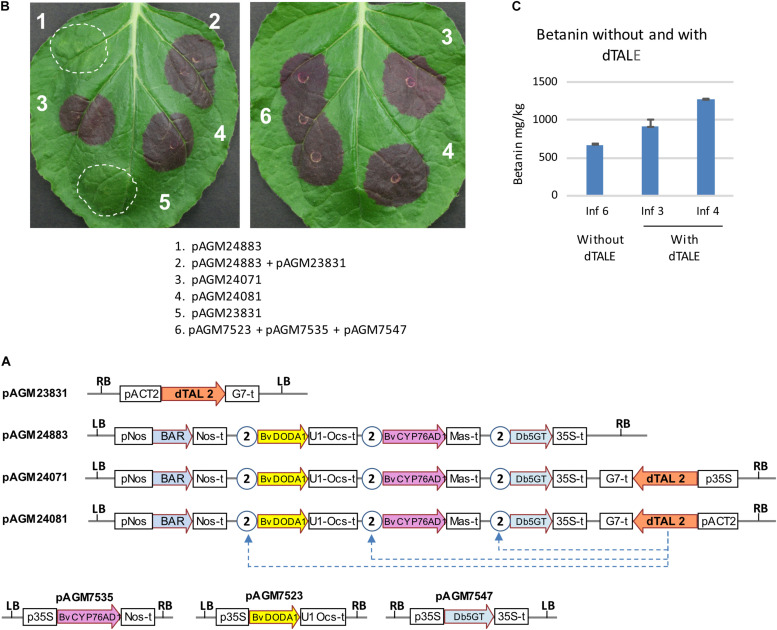
Transient expression of dTALE constructs. **(A)** Structure of the constructs infiltrated in the benthamiana leaves shown in (B). P35S, 35S promoter; Nos-t, Nos terminator; U1 Ocs-t, 3′ untranslated sequences from TMV U1 viral genome followed by Ocs terminator; 35S-t, 35S terminator; Mas-t, Mas terminator; G7-t, G7 terminator; pACT2, Arabidopsis ACT2 promoter; LB and RB, left and right T-DNA borders. The STAP promoter 2, shown as circles with the number 2, contain a binding site for the dTALE 2 protein. **(B)** Transient infiltration in *N. benthamiana* leaves. The description of the combination of constructs infiltrated in the leaves (infiltrations 1–6) are shown below. The pictures were taken 5 days after infiltration. **(C)** Quantification of betanin in the infiltrated leaf areas 3, 4, and 6 of the leaf shown on the right in **(B)**.

To express all genes of the pathway at the same time, we made constructs that rely on transcriptional activation of pathway genes by a dTALE. We used a dTALE (dTAL2) that was previously made and that activate transcription by binding to an 18 bp sequence in a promoter (Weber et al., 2011). We cloned the dTALE under control of the Arabidopsis *ACT2* or 35S promoters, as we first wanted to test the constructs by transient expression in *N. benthamiana* leaves. Several constructs were made with the dTALE and the betalains biosynthetic genes cloned either on separate T-DNAs or on the same T-DNA (Figure 3A). The same STAP promoter was used for all pathway genes, as its small size (97 bp) was unlikely to induce construct instability. Expression of the dTALE construct (with the *ACT2* promoter) alone did not lead to any visible phenotype in the infiltrated area (Figure 3B, infiltration 5). Infiltration of the construct with all genes of the pathway under control of STAPs (pAGM24883 and pAGM24895) but without the dTALE also did not lead to any visible betanin production (Figure 3B, infiltration 1). This shows that the STAPs have no background transcriptional activity in the absence of a corresponding dTALE. When both constructs were coinfiltrated (the dTALE construct pAGM23831 and the biosynthetic genes under control of STAP promoters, pAGM24883), strong betanin biosynthesis was observed (Figure 3B, infiltration mix 2). Finally, infiltration of constructs with all pathway genes and the dTALE on the same T-DNA (pAGM24071, with dTALE under control of a 35S promoter, and pAGM24081, with the dTALE under control of the ACT2 promoter) led to betanin biosynthesis (infiltrations 3 and 4). Betanin production obtained by co-infiltration of the three constructs with pathway genes under control of the 35S promoter was compared with production obtained by infiltration of all-in-one constructs containing the dTALE and all pathway genes (Figure 3B, infiltrations 6, 3, and 4). The amount of betanin in the same leaf increased from 670 mg/kg for constructs with the 35S promoter to 900 and 1,200 mg/kg for constructs with dTALEs with the *ACT2* or 35S promoters, respectively (Figure 3C).

**FIGURE 4:**
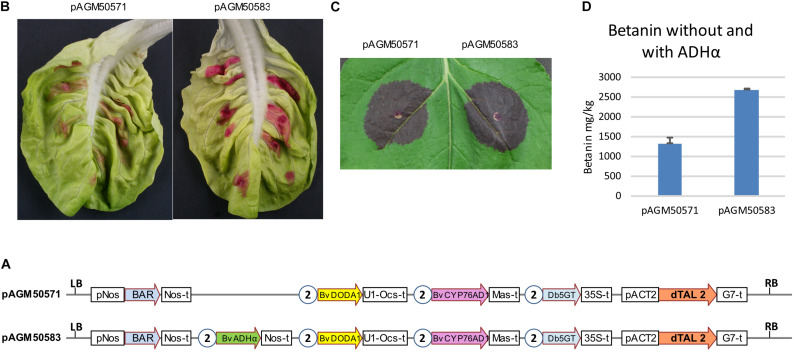
Transient expression of dTALE constructs without or with ADHα. **(A)** Structure of the constructs infiltrated in leaves shown in **(B,C)**. pAGM50571 differs from pAGM50583 by the presence of the ADHα coding sequence in pAGM50583. Same legend as in Figure 3. **(B,C)** Transient infiltration in lettuce **(B)** and *N. benthamiana* leaves **(C)**. Pictures taken 5 days after infiltration. **(D)** Quantification of betanin in the *N. benthamiana* leaf shown in **(C)**.

To test the effect of ADHα with all genes in one T-DNA, two constructs were made, one with all three genes for betanin biosynthesis and the dTALE (pAGM50571), and a second with the addition of the ADHα gene (pAGM50583) (Figure 4A). Both constructs were infiltrated in lettuce leaves or *N. benthamiana* (Figures 4B,C, respectively). In both cases, constructs with the ADHα led to stronger purple color, suggesting increased betanin biosynthesis. Betanin levels in the *N. benthamiana* leaf indeed increased from 1,300 mg/kg without ADHα to 2,700 mg/kg with ADHα (Figure 4D).

### Tomato Transformation

Our goal was to generate tomato plants in which the betanin biosynthesis genes would be expressed in fruits only. We used the E8 promoter, which is active in fruits at the time of ripening (Deikman et al., 1998). This promoter has been previously shown to work well for expression of transcription factors for anthocyanin biosynthesis in tomato fruits (Butelli et al., 2008).

**FIGURE 5:**
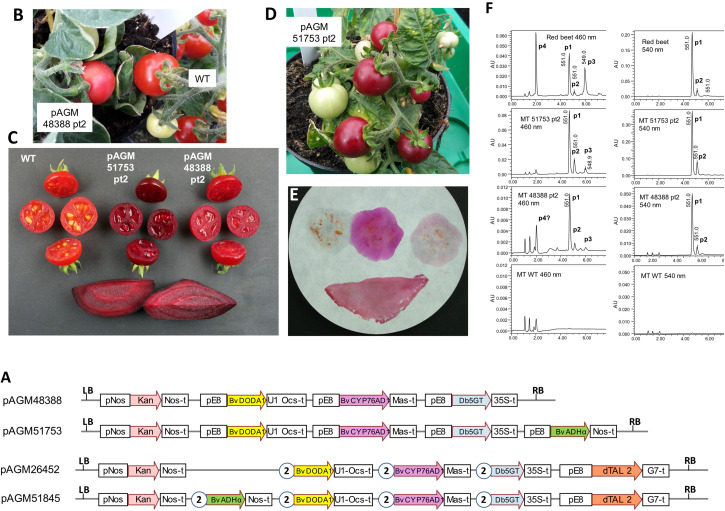
Betalain biosynthesis by stable transformation of Micro Tom tomatoes. **(A)** Structure of the level 2 constructs for tomato transformation. pE8, fruit-specific tomato E8 promoter. All other legends as in Figure 3. **(B)** Micro Tom pAGM48388 transformant 2. **(C)** Pictures of fruits of WT Micro Tom, pAGM51753 transformant 2, pAGM48388 transformant 2, and red beet slices as a control. **(D)** Micro Tom pAGM51753 transformant 2. **(E)** Fruits cut in half shown in **(C)** were blotted on filter paper in the order, from left to right, upper section: WT, pAGM51753 and pAGM48388. The lower part shows the blotting of a red beet slice. **(F)** LC/MS analysis of the extracts of fruits pAGM48388 and pAGM51753 transformants and of WT Micro Tom, and of a red beet as control. P1, betanin; P2, isobetanin, P3, neobetanin; P4, vulgaxanthin I. Note that the scale range (AU) is not the same for all samples.

We first made constructs with all pathway genes under control of a STAP promoter and a corresponding dTALE under control of the E8 promoter (Figure 5A). Two constructs were made, one containing the 3 genes for betalain biosynthesis but not the ADHα gene (pAGM26452), and a second containing all 4 genes (pAGM51845). Unfortunately, no transformant producing betanin in fruits could be obtained with these constructs.

We therefore made two constructs with all pathway genes under control of the E8 promoter, one construct lacking the ADHα gene (pAGM48388) and one containing it (pAGM51753). The version of the E8 promoter that we used is 2.16 kb long, and therefore, cloning this sequence 3 or 4 times in the same construct may lead to construct instability. The constructs could nevertheless be cloned in *E. coli* strain DH10B, and plasmid miniprep DNA was transformed into Agrobacterium strain GV3101. To minimize the chance of recombination between repeated sequences in Agrobacterium, a procedure was used to reduce the number of Agrobacterium cell divisions until the cultures were used for transformation of tomato cotyledon tissue. This procedure consisted of transforming the constructs in Agrobacterium competent cells by electroporation and plating the entire transformation on a single selection plate. This gave rise to a lawn of bacteria rather than individual colonies. A loop of Agrobacterium taken from the plate was then directly used for inoculation of medium for plant transformation.

Transformation of pAGM48388 led to regeneration of 8 transformants. Only one of these, pAGM48388 primary transformant 2 (pAGM48388 pt2) had fruits with a slightly different color than the WT control, but this difference was minor (Figure 5B). The color difference was, however, more visible after cutting the fruits in half (Figure 5C). The presence of all three genes of the betalain biosynthesis pathway in this plant was confirmed by PCR amplification from genomic DNA (Supplementary Figure 7). Of the remaining 7 plants, 6 had lost the first two genes of the pathway, and one plant was probably not transformed (pAGM48388 primary transformant 5). The fruits of pAGM48388 transformant 2 contained a few seeds, but in a lower amount in comparison to a WT fruit.

Transformation of pAGM51753 led to regeneration of 8 transformants. Three of these transformants (primary transformants 2, 7, and 8; pAGM51753 pt2, pt7, and pt8) produced fruits with dark purple/burgundy color, suggesting betanin biosynthesis (Figure 5D). Transformant 8 was a very small plant with only one fruit that did not make seeds. The two other transformants, pt2 and pt7, had fruits that set seeds normally. Except for the fruit phenotype, all other tissues of these three plants had a normal WT phenotype, as expected from the fruit-specific expression of the E8 promoter. Analysis of genomic DNA of transformants 2 and 7 by PCR showed that all 4 genes of the constructs were indeed present in these plants (Supplementary Figure 8). The remaining 5 transgenic plants had fruits with a normal WT phenotype. Two of 3 analyzed plants contained the ADHα gene, and one contained in addition the glucosyltransferase gene. One plant contained only the kanamycin selection marker.

The color of pAGM51753 pt 2, 7, and 8 was much stronger than pAGM48388 pt 2, suggesting a much higher betalain content. The color of the fruits cut in half was similar to the color of a slice of red beet (Figure 4C). The cut fruits were blotted on filter paper. For a control Micro Tom WT fruit, no color was visible on the paper, except a little bit of color that came from fragments of tissue left on the filter, which contained carotenoids (Figure 5E). Blotting of a fruit of pAGM48388 pt 2 produced weak purple color. In contrast, blotting of a fruits from pAGM51753 pt 2 produced very strong fuchsia color, suggesting the presence of a relatively high amount of betanin. The color of the juice blotted on filter paper was different from the color of the fruits. This can be explained by the fact that the color from fruits comes from betanin and carotenoids while the liquid blotted on paper contains betanin only. Interestingly, blotting of a slice of red beet produced a different color compared to the pAGM51753 transformant, a color that can be described as burgundy. This color may be explained by the presence of betacyanins (betanin) and betaxanthins in red beet juice, while the juice of the tomato transformants produce mostly betanin (see next paragraph).

### Analysis of Betalain Production in Tomato Fruits

Extracts from tomato transformants and from a red beet control were analyzed by LC/MS (Figure 5F). At 540 nm, extracts of fruits from pAGM48388 pt2 and pAGM51753 pt2 revealed a main peak of betanin and a small peak of isobetanin. The amount of betanin and isobetanin was, however, much smaller from pAGM48388 pt2 than from pAGM51753 pt2, explaining the difference in color. As mentioned above, red beet extracts displayed 4 peaks identified as betanin, isobetanin, neobetanin, and vulgaxanthin I. At 560 nm, fruits of the pAGM51753 transformant 2 also showed a small peak of a betaxanthin, identified as vulgaxanthin I based on UV- and MS-data, that overlapped with a background peak from tomato. The same compound may have been present in the pAGM48388 transformant, but in very low amount as total betalains were much lower in this transformant.

**FIGURE 6:**
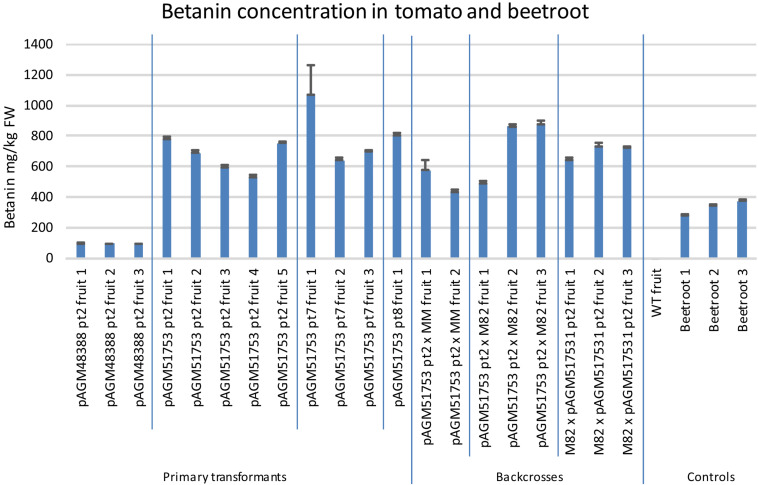
Betanin concentration in tomato transformants and in beetroot. Betanin was quantified in several fruits harvested from the primary tomato transformants or from plants obtained from the backcrosses of pAGM51753 transformant 2 with Moneymaker or M82. The fruits were harvested when they were ripe, and were not all harvested on the same date. In addition, a fruit with WT phenotype (lacking betalain) obtained from the M82 × pAGM51753 cross was used a WT control. Betanin content of three beetroots were also measured. For all samples, quantification was done in triplicate from three aliquots of tissue from the same fruit or beetroot. The error bars show the standard deviation of the three measurements.

The betanin content in the tomato transformants was also quantified by UV/Vis spectroscopy (Figure 6, pictures of some of the extracts in Supplementary Figure 9). Fruits of pAGM48388 pt2 transformant (without ADHα) had a betanin content of about 94 mg/kg. In contrast fruits of the 3 pAGM51753 transformants (with ADHα) had a betanin content between 530 and 1,000 mg/kg, with an average of 680 mg/kg for fruits of pAGM51753 pt2 transformants.

### Analysis of the Insertion Site of pAGM51753 Transformant 2

The T-DNA insertion site in the tomato genome was identified by PCR amplification of flanking T-DNA sequences and DNA sequencing. A complete T-DNA is inserted in the UTR of gene Solyc05g007230, 1.8 kb upstream of the ATG start codon. This is within the UTR, which is 2.4 kb long (Supplementary Figure 10). The T-DNA insertion resulted in a 56 bp deletion at the insertion site. This gene is a Leucine Rich Repeat receptor-like serine/threonine-protein kinase called GSO1. Two Arabidopsis homologues *GASSHO1* and *GASSHO2* are involved in formation of the epidermal surface during embryogenesis. Homozygous double mutant Arabidopsis plants have abnormal phenotypes, but plant mutant for only one of either gene have no visible phenotype (Tsuwamoto et al., 2008). Interestingly, selfed progeny of pAGM51753 transformant 2 were analyzed by PCR using primers flanking the insertion and one T-DNA primer. Out of 7 plants grown, only one plant had a WT phenotype and 6 plants had the betalain phenotype. However, all 6 plants were heterozygous (Supplementary Figure 11). Screening of an additional fifteen seedlings that were randomly selected for DNA extraction before they produced fruits showed that 14 were heterozygous for the T-DNA insertion and one was homozygous WT (Supplementary Figure 11).

### Backcrosses to Tomato Cultivar M82 and Moneymaker

**FIGURE 7:**
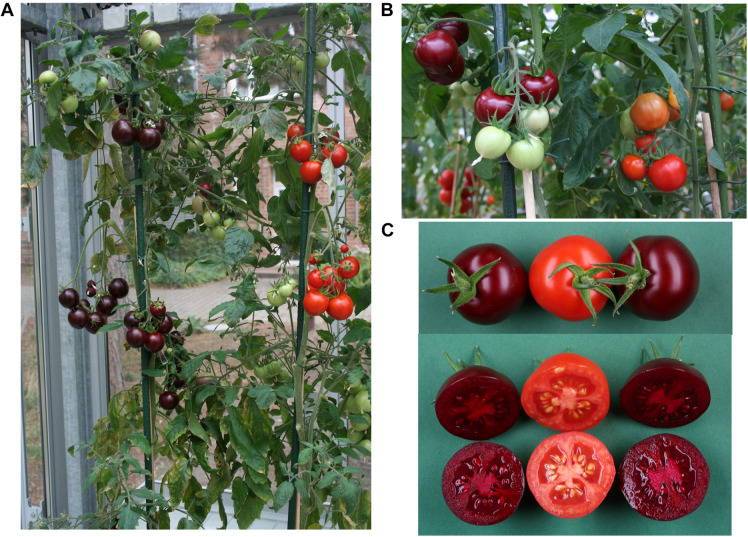
Pictures of tomatoes from the backcross of M82 × pAGM51753 pt2. Sibling plants from the same cross, with or without the T-DNA are shown, with betalains (purple fruit) and without (orange fruits). Plants with fruits at different stages of ripening are shown, with inflorescences with all fruits ripe **(A)** or not all fruits ripe **(B)**, showing that betalains are produced only at the fruit ripening stage. **(C)** Fruits from siblings with or without betalain biosynthesis.

The selfed progeny of pAGM51753 transformant 2 was backcrossed several times to tomato cultivars M82 and Moneymaker, up to backcross 3 in cultivar Moneymaker and backcross 4 in cultivar M82 (Supplementary Figures 12, 13). The plants are not dwarf anymore as is Micro Tom. Plants of the BC1 in M82 are shown in Figure 7. The color of the fruits of any of the backcrosses was still as high as in the original transgenic plant, and was uniform in all fruits of the plant. This shows that having the E8 promoter repeated 4 times in the construct does not lead to construct instability once it is transformed into plants. Betanin levels in the crosses were in the same range as in the primary transformants (Figure 6). Some variation in the fruits of both primary transformants and of the crosses probably comes from the fact that the fruits collected did not all have exactly the same age.

### Use of the Tomato Juice as Food Colorant

**FIGURE 8:**
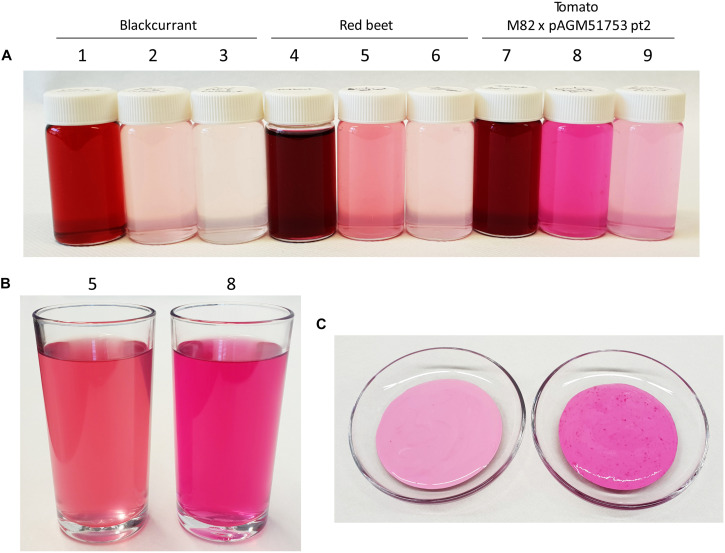
Use of betalain-containing tomato juice as food colorant. **(A)** Preparation of lemonade colored with blackcurrant juice, red beet or betanin-producing tomatoes. The composition of the bottles in **(A)** is as follow: (1) 4.3 g juice + 50 g sugar/100 ml, (2) 0.26 g juice + 3 g sugar + 3 ml lemon juice/100 ml, (3) 0.09 g juice + 3 g sugar + 3 ml lemon juice/100 ml, (4 and 7) 8.6 g ground tissue + 50 g sugar/100 ml, (5 and 8) 0.52 g ground tissue + 3 g sugar + 3 ml lemon juice/100 ml, (6 and 9) 0.17 g ground tissue + 3 g sugar + 3 ml lemon juice/100 ml. **(B)** same preparation as for (5, red beet) and (8, betanin tomato) from **(A)** but in a larger volume. **(C)** 30 g of yogurt colored by addition of 3 g of red beet juice (left picture) or betanin tomato juice (right picture).

The intense color of the juice of betanin-producing tomatoes suggests that it could be used as food colorant. To test this possibility, lemonade was prepared using the juice of pAGM51752 pt2 progeny plants by adding 0.5 g of tomato fruit ground tissue, 3 g of sugar, 3 ml of lemon and water to a final volume of 100 ml. A diluted lemonade version was made containing 3 times less ground tissue for the same amount of sugar and lemon juice. For comparison, the same recipes were used with ground red beet and blackcurrant. Since only blackcurrant juice was available, 4.3 g of juice was used instead of 8.6 gram of ground tissue. The lemonade made with betanin-producing tomatoes had a bright purple-fuschia color (Figures 8A,B, flask or glass 8). In comparison, the red beet and blackcurrant lemonades had a less bright and more red-brown color.

The betanin-producing tomatoes and red beet were also used to color yogurt. Three g of juice squeezed from red beet or tomatoes were added to 30 g of yogurt. This led to a strong red-purple color with the tomato juice, and a less intense color with the red beet juice (Figure 8C).

### Cloning of More Stable Constructs in Low Copy Plasmids

**FIGURE 9:**
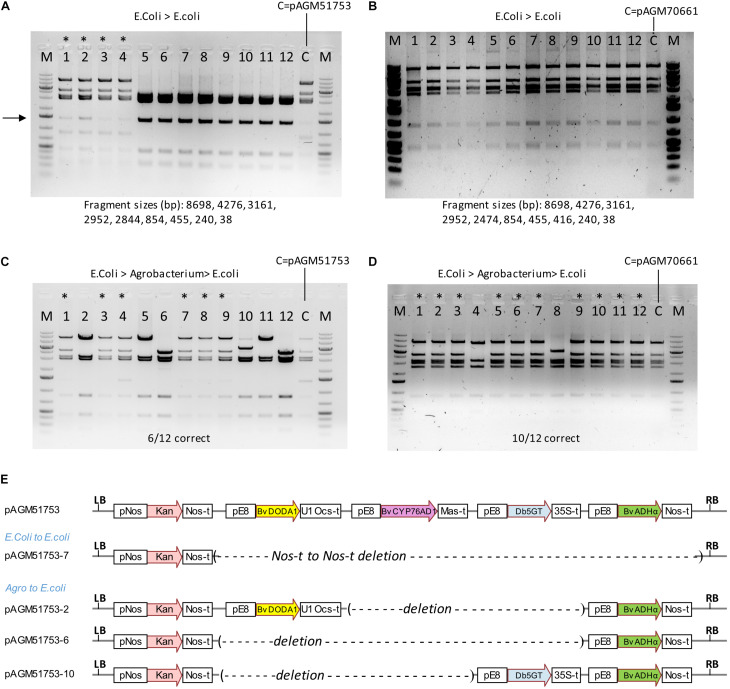
Stability of constructs in high and low copy plasmid backbones. **(A)** Digests with NheI and SpeI of DNA of 12 minipreps made from colonies obtained after retransformation of the initial plasmid (pAGM51753) in *E. coli*. The original plasmid prep was digested and shown as a control (c). The first 4 minipreps were from small colonies, while the 8 others were from larger colonies. A DNA fragment of variable intensity (shown by an arrow) is visible in digests from minipreps 1–4, and from the miniprep from the initial plasmid. This fragment is the result of a recombination event in *E. coli*. **(B)** Same as in **(A)** with the low copy plasmid pAGM70661. In this case, all colonies had the correct size. **(C)** pAGM51753 plasmid was transformed in Agrobacterium strain GV3101. Plasmid DNA extracted from transformed agrobacterium cells was retransformed in *E. coli*. The restriction digest of 12 randomly picked colonies shows 6 colonies with the correct pattern (labeled with a star), and 6 colonies with recombined DNA. **(D)** Same as in **(C)** with construct pAGM70661. Digests with NheI and SpeI of plasmid DNA shows that 10 out of 12 colonies have plasmids identical to the starting non-recombined plasmid (labeled with a star). **(E)** Structure of construct pAGM51753 and of recombined plasmids pAGM51753-7 from **(A)** and pICH51753-2, 6, and 10 from **(C)**.

We succeeded in making transgenic tomato plants with a construct containing 4 times the same 2.16 kb promoter fragment. Transformation of cultivar Micro Tom was successful, but a later transformation attempt directly in cultivar Moneymaker was not. It is likely that recombination between repeats is the reason why we could not regenerate plants with a complete construct. These recombination events could have occurred in *E. coli*, Agrobacterium, during T-DNA integration in plants or later in transgenic plants. Since the cloning vector of the level 2 constructs replicates at high copy in *E. coli*, propagation in *E. coli* is a likely place for recombination events. A digest of pAGM51753 miniprep DNA showed the presence of the expected restriction fragments, but also of a DNA fragment of unexpected size of weaker intensity (shown with an arrow in Figure 9A). This may result from a recombination event that occurred in some *E. coli* cells that initially contained the complete correct construct. This may have led to the presence, within the same colony, of different subsets of cells that contained the original complete or the recombined plasmid. Plasmid miniprep DNA was retransformed into *E. coli*. The transformation plate revealed the presence of colonies of different sizes. Four colonies of sizes small, medium and large were grown and DNA extracted. DNA digestion reveals that the 4 small colonies contained the correct construct while all other colonies contained recombined plasmids (Figure 9A). Sequencing one of the recombined clones revealed a recombination event between the Nos terminator of the Kanamycin selectable marker and the Nos terminator of the ADHα transcription unit, resulting in deletion of the 4 betalain biosynthesis genes (Figure 9E). The 4 DNA digests with the correct constructs also had the additional band seen in the original miniprep. This fragment varied in intensity in the different digests, consistent with a randomly occurring recombination event in cells of the colonies. Retransformation of another miniprep of the construct pAGM51753 (called pAGM51751, but with exactly the same sequence) resulted in 6 colonies out of 12 containing the non-recombined construct (Supplementary Figure 14).

To analyze construct stability in Agrobacterium, plasmid DNA of pAGM51753 and pAGM51751 was transformed in Agrobacterium GV3101. Plasmid DNA was prepared from both Agrobacterium cultures, and retransformed in *E. coli* for analysis. Plasmids minipreps were made from 12 randomly selected *E. coli* colonies for both retransformations. For retransformed clones derived from pAGM51753, 6 had a restriction pattern identical to the initial non-recombined plasmid, and that 6 had digests showing different types of recombination events (Figure 9C). The presence of some non-recombined clones explain why we were able to generate transgenic tomato plants containing the complete construct. Sequencing of plasmid of three colonies with different digestion patterns (pAGM51753-2, 6, and 10) indicated recombination events between E8 promoter repeats, leading to loss of 2 or 3 betalain biosynthesis genes (Figure 9E). All 12 colonies obtained by retransformation from Agrobacterium cultures to *E. coli* from pAGM51751 contained only recombined plasmids (Supplementary Figure 14).

To try to generate more stable constructs, a construct similar to pAGM51753 was cloned in a plasmid backbone that contains the p15a origin of replication (ori) for medium/low copy replication in *E. coli* and the *Agrobacterium rhizogenes* A4 ori for single copy replication in Agrobacterium. A miniprep of this construct, pAGM70661, showed the presence of the expected restriction fragments only. This plasmid was retransformed in *E. coli*, and minipreps prepared from the resulting colonies were analyzed by restriction digest. All 24 colonies tested contained plasmids with the correct digestion pattern (the first 12 are shown in Figure 9B). Retransformation of another miniprep of construct pAGM70661 (called pAGM70666, but with the same sequence) also resulted in 12 colonies out of 12 containing a non-recombined construct (Supplementary Figure 15). This shows that a construct with the p15a ori backbone is stable in *E. coli*, even with a 2.2 kb DNA fragment present 4 times. Plasmid DNA from pAGM17006 and pAGM70666 was transformed in agrobacterium strain GV3101. Plasmid DNA was prepared from both Agrobacterium cultures, and retransformed in *E. coli* for analysis. Analysis of 12 colonies from each transformation showed that 10 out of 12 and 12 out of 12 colonies contained the non-recombined plasmid (Figure 9D and Supplementary Figure 15). Therefore, use of the construct in the low copy backbone is more stable in *E. coli* and results in more Agrobacterium cells containing the non-recombined construct.

## Discussion

We have tested expression of three genes involved in betanin biosynthesis using transient assays in *N. benthamiana* leaves. The cytochrome P450 and the L-DOPA dioxygenase had previously been tested and used to reconstruct a betanin biosynthesis pathway in heterologous hosts (Hatlestad et al., 2012; Polturak et al., 2016, 2017), but not the *D. bellidiformis* glucosyltransferase. We have shown here that this enzyme works as a cyclo-DOPA glucosyltransferase in addition to its previously identified activity as a betanidin glucosyltransferase (Vogt et al., 1999). Expression of the first two genes of the pathway, BvCYP76Ad1 and BvDODA1 without a glucosyltransferase should have led to biosynthesis of betanidin. Betanidin was not detected, but was probably made and quickly degraded as it is unstable. Interestingly, LC/MS analysis of the extracts obtained by co-expression of *BvCYP76Ad1* and *BvDODA1* revealed a peak with the same mass as betanin but a different retention time. This peak was identified as gomphrenin I, as it has the same mass and retention time as a purified standard from *G. globosa* (Heuer et al., 1992). Gomphrenin I is the positional isomer of betanin and is synthesized by glucosylation of the 6-OH position of betanidin, instead of the 5-OH group for biosynthesis of betanin. Gomphrenin I was likely made by the non-specific activity of a *N. benthamiana* glucosyltransferase. Overlapping glucosyltransferase specificities for flavonols and betalains have been reported previously from *D. bellidiformis* (Vogt et al., 1997). Recombinant *D. bellidiformis* betanidin 6-GT purified enzyme preferentially catalyzed the formation of quercetin-3-*O*-glucoside from quercetin *in vitro*, whereas *D. bellidiformis* 5GT catalyzed the formation of quercetin 4′-O-glucoside. Since flavonoid 3-*O*-glycosylation is the most common modification in flavonol biosynthesis, it is plausible that an endogenous *N. benthamiana* 3-*O*-glucosyltransferase produced gomphrenin I, the 6-O-glucoside, rather than betanin, the 5-O-glucoside. The amount of gomphrenin I made in *N. benthamiana* was, however, much lower than the amount of betanin made when all three betanin biosynthesis genes were expressed. This peak was also not seen in infiltrations where a betanidin 5GT glucosyltransferase or cyclo-DOPA glucosyltransferase was available, suggesting that expression levels of the Db5GT was sufficient for a complete turnover to betanin, and only traces of cyclo-DOPA “escape” toward the spontaneous betaxanthin, i.e., vulgaxanthin I formation. LC/MS analysis of the BvCYP76Ad1 and BvDODA1 co-expression extract also revealed a small peak that may contain traces of betanin as a result from the weak non-specific activity of another promiscuous *N. benthamiana* enzyme.

Betanin was produced when all three betanin biosynthesis genes were expressed. Interestingly, the amount of betanin produced in *N. benthamiana* was surprisingly high, at about 700 mg/kg of fresh weight, which is about two times more than betanin extracted from fresh red beets. The beets that were used for these measures were from an unknown cultivar (purchased in a supermarket), but had the typical color of normal beetroots. It is nevertheless possible that other red beet cultivars may have higher betanin content, as betalain concentrations have been reported to vary from 97 mg of betacyanins/100 ml to 500 mg betanin/kg of fresh weight (Lee et al., 2014; Skalicky et al., 2020). By coexpression of ADHα with the three other genes, the amount of betanin reached 2.3–2.7 g/kg of fresh weight, about 4 times the amount found in the red beet control. The high amount of betanin made in *N. benthamiana* leaves is probably the result of a high tyrosine biosynthesis capability of *N. benthamiana* leaves, the use of the ADHα gene to boost tyrosine production, and the composition of the level 1 expression constructs that were optimized for high level expression of the biosynthesis genes (as described in the section “Materials and Methods”).

Expressing biosynthetic pathways in heterologous hosts requires expressing all genes of the pathway at the same time and in the same tissue. We have shown here in transient assays in *N. benthamiana* leaves that the use of dTALE constructs can result in a high level of expression of all genes of the pathway. The expression level was slightly higher than when all genes of the pathway were expressed from the 35S promoter. Unfortunately, dTALE constructs could not be transformed in tomato plants, and one explanation is that TAL effectors may have induced an immune response in tomato plants due to the presence of the Bs4 gene (Schornack et al., 2005). One may have expected that the absence of expression of the dTALE in regenerating plants, due to fruit-specificity of the E8 promoter, should have prevented any potential negative effect on regeneration of transgenic plants. However, it is possible that even low expression of the dTALE in the regenerants may have been enough to prevent the production of plants with complete constructs. The use of dTALE constructs should nevertheless be possible in other species, and we have successfully transformed other dTALE constructs unrelated to this work in Arabidopsis (not shown). For use in tomato, replacing the dTALE with a CRISPR transcriptional activator would be a solution (Li et al., 2017; Selma et al., 2019).

Since TAL effector constructs could not be used for tomato transformation, and to avoid the risk of low gene expression by using a 2A fusion strategy, we decided to make constructs with all genes of the pathway cloned with the same promoter. An obvious drawback of this strategy is that the repeats in the construct may lead to instability in *E. coli*, Agrobacterium or in the plant after transformation. In this work we have shown that constructs with a 2.2 kb promoter repeated 4 times could be cloned and used for plant transformation. The constructs obtained were, however, unstable in *E. coli* and Agrobacterium. Nevertheless, these constructs could be transformed in plants by taking precautions to limit the number of cell divisions to which the *E. coli* and Agrobacterium strains were exposed to before transformation. We were able to regenerate three transformants (out of 8 transformants) that expressed all genes of the pathway and produced betalains at high level. One transformant was characterized at the molecular level (pAGM51753 transformant 2), and the presence of a single, apparently complete, T-DNA was found. The transformants that did not produce betalains lacked some of the genes of the biosynthetic pathway, and it is likely that recombination between repeated sequences of the E8 promoter occurred during passage in *E. coli*, Agrobacterium, or during integration of the T-DNA in the plant genome. Interestingly, the phenotype of the transgenic plants or their progeny was extremely stable. We made 3 or 4 backcrosses of the pAGM51753 transformant 2 with cultivars Moneymaker and M82, respectively, and did not observe a loss of color and/or a loss of betanin production level in any of these progenies. This suggests that once integrated on the chromosome, the risk of deletion between repeats is extremely unlikely. This is not entirely surprising as the genome of most plants contain large numbers of repeated sequences (transposons and retrotransposons) but are nevertheless stable. Even if transformation of a construct containing the same promoter multiple times is successful, another problem that one might expect is that the genes may become transcriptionally or post-transcriptionally silenced. In the present example, no sign of silencing could be detected in the transformants or the progeny of these transformants, for several generations. Silencing of the constructs would have led to a phenotype with fruits with a different color (less purple and more orange-red as normal tomatoes), fruits with sectors of different color or with irregular coloration, or the presence of some fruits with and without color on the same plant.

While the strategy that uses the same promoter multiple times was successful, obtaining transformants with a complete construct was difficult. For example, we also tried to directly transform construct pAGM51753 in cultivar Moneymaker, but none of the transformants displayed betalain production in fruits. However, we show here that cloning the same construct in a low copy vector (with the p15a ori for replication in *E. coli*) significantly reduces the instability of the construct in *E. coli*. In fact, not a single recombination event could be detected in 24 analyzed *E. coli* colonies obtained by retransformation of DNA of the initial miniprep. The higher stability in *E. coli* led to an increase in the number of Agrobacterium cells containing non-recombined construct, and should increase the chance of obtaining transformed plants containing the non-recombined construct. The low copy vector could be useful for making other constructs that would contain repeated sequences, and has therefore been deposited to Addgene^1^.

The tomato transformants expressing all three genes for betanin biosynthesis, but not the *ADHα*, produced betanin at a relatively low level. Expressing an *ADHα* gene in addition of the three betanin biosynthesis genes led to a dramatic increase of betanin biosynthesis of about 10-fold. This corresponds to the increase in tyrosine production that was previously observed by expression of the *ADHα* gene in *N. benthamiana* leaves (Lopez-Nieves et al., 2018). The amount of betacyanins (betanin and isobetanin) present in tomato fruits was higher than what is found in the red beets used as control. Interestingly, the color of the juice of these tomatoes had a bright fuchsia red color, unlike the more burgundy red color of red beet juice (Supplementary Figure 9). This is because red beets produce betanin and isobetanin, as well as neobetanin, which has an orange/yellow color, and betaxanthins that have a yellow color, while the engineered tomato produced mostly betanin and isobetanin. The unique color from the juice of the engineered tomatoes and the high amount produced in fruits provides an interesting source for a bright fuchsia color food colorant.

We have therefore shown that engineering a biosynthetic pathway in transgenic plants using the same tissue-specific promoter several times can be a viable solution. The use of transcription factors and of associated promoter libraries nevertheless remains the solution of choice for precise engineering of biosynthetic pathways in heterologous hosts.

## Data Availability Statement

The raw data supporting the conclusions of this article will be made available by the authors, without undue reservation.

## Author Contributions

RG, CH, and CY performed the research. RS generated tomato transformants and made tomato backcrosses. TV contributed to the betalains LC/MS analysis and analyzed data. SM designed the experiments and wrote the article with the contribution of TV and all authors.

## Conflict of Interest

The authors declare that the research was conducted in the absence of any commercial or financial relationships that could be construed as a potential conflict of interest.

## Footnotes

1
https://www.addgene.org/

## Supplementary Material

Supplementary Figure 1 Sequence of the level 0 modules for BvCYP76AD1, BvDODA1, Db5GT, BvADHα, dTAL2, and STAP2.

Supplementary Figure 2 List of parts and vectors for cloning level 1 constructs for transient expression and level 2 constructs for tomato stable transformation.

Supplementary Figure 3 List of vectors and parts deposited at Addgene.

Supplementary Figure 4 Expression of BvCYP76AD1 and BvDODA1 in *Nicotiana benthamiana* leaves results in gomphrenin biosynthesis.

Supplementary Figure 5 Expression of BvCYP76AD1 and BvDODA1 in *Nicotiana benthamiana* leaves results in low amount of betaxanthins.

Supplementary Figure 6 Quantification of betanin production by transient expression in *Nicotiana benthamiana* leaf with and without ADHα.

Supplementary Figure 7 PCR analysis of the T-DNAs of pAGM48388 transformants.

Supplementary Figure 8 PCR analysis of the T-DNAs of pAGM51753 transformants.

Supplementary Figure 9 Extracts from *N. benthamiana* leaves, red beets and tomatoes.

Supplementary Figure 10 Analysis of the T-DNA insertion site in pAGM51753 transformant 2.

Supplementary Figure 11 PCR analysis of selfed progenies of pAGM51753 pt2.

Supplementary Figure 12 List of backcrosses of pAGM51753 transformant 2 with tomato cultivars Moneymaker and M82.

Supplementary Figure 13 Fruits obtained after 3 backcrosses with M82 or Moneymaker.

Supplementary Figure 14 Plasmid stability of high copy plasmids in *E. coli* and Agrobacterium.

Supplementary Figure 15 Plasmid stability of low copy plasmids in *E. coli* and Agrobacterium.

## References

[B1] Alard D. Wray V. Grotjahn L. Reznik H. Strack D. (1985). Neobetanin - isolation and identification from beta-vulgaris. *Phytochemistry* 24 2383–2385. 10.1016/s0031-9422(00)83046-0

[B2] Belcher M. S. Vuu K. M. Zhou A. Mansoori N. Agosto Ramos A. Thompson M. G. (2020). Design of orthogonal regulatory systems for modulating gene expression in plants. *Nat. Chem. Biol.* 16 857–865. 10.1038/s41589-020-0547-4 32424304

[B3] Boch J. Scholze H. Schornack S. Landgraf A. Hahn S. Kay S. (2009). Breaking the code of DNA binding specificity of TAL-Type III effectors. *Science* 326 1509–1512. 10.1126/science.1178811 19933107

[B4] Bruckner K. Schafer P. Weber E. Grutzner R. Marillonnet S. Tissier A. (2015). A library of synthetic transcription activator-like effector-activated promoters for coordinated orthogonal gene expression in plants. *Plant J. Cell Mol. Biol.* 82 707–716. 10.1111/tpj.12843 25846505 PMC4691316

[B5] Butelli E. Titta L. Giorgio M. Mock H. P. Matros A. Peterek S. (2008). Enrichment of tomato fruit with health-promoting anthocyanins by expression of select transcription factors. *Nat. Biotechnol.* 26 1301–1308. 10.1038/nbt.1506 18953354

[B6] Cai Y. M. Kallam K. Tidd H. Gendarini G. Salzman A. Patron N. J. (2020). Rational design of minimal synthetic promoters for plants. *Nucleic Acids Res.* 48 11845–11856. 10.1093/nar/gkaa682 32856047 PMC7708054

[B7] Deikman J. Xu R. Kneissl M. L. Ciardi J. A. Kim K. N. Pelah D. (1998). Separation of cis elements responsive to ethylene, fruit development, and ripening in the 5′-flanking region of the ripening-related E8 gene. *Plant Mol. Biol.* 37 1001–1011. 9700072 10.1023/a:1006091928367

[B8] Engler C. Youles M. Gruetzner R. Ehnert T. M. Werner S. Jones J. D. (2014). A golden gate modular cloning toolbox for plants. *ACS Synthetic Biol.* 3 839–843. 10.1021/sb4001504 24933124

[B9] Esatbeyoglu T. Wagner A. E. Schini-Kerth V. B. Rimbach G. (2015). Betanin–a food colorant with biological activity. *Mol. Nutr. Food Res.* 59 36–47. 10.1002/mnfr.201400484 25178819

[B10] Gandia-Herrero F. Escribano J. Garcia-Carmona F. (2016). Biological activities of plant pigments betalains. *Crit. Rev. Food Sci. Nutr.* 56 937–945. 10.1080/10408398.2012.740103 25118005

[B11] Hatlestad G. J. Sunnadeniya R. M. Akhavan N. A. Gonzalez A. Goldman I. L. McGrath J. M. (2012). The beet R locus encodes a new cytochrome P450 required for red betalain production. *Nat. Genet.* 44 816–820. 10.1038/ng.2297 22660548

[B12] He Y. Zhang T. Sun H. Zhan H. Zhao Y. (2020). A reporter for noninvasively monitoring gene expression and plant transformation. *Horticult. Res.* 7:152. 10.1038/s41438-020-00390-1 33024566 PMC7502077

[B13] Heuer S. Wray V. Metzger J. W. Strack D. (1992). Betacyanins from flowers of gomphrena-globosa. *Phytochemistry* 31 1801–1807. 10.1016/0031-9422(92)83151-n

[B14] Kay S. Hahn S. Marois E. Hause G. Bonas U. (2007). A bacterial effector acts as a plant transcription factor and induces a cell size regulator. *Science* 318 648–651. 10.1126/science.1144956 17962565

[B15] Lee E. J. An D. Nguyen C. T. Patil B. S. Kim J. Yoo K. S. (2014). Betalain and betaine composition of greenhouse- or field-produced beetroot (*Beta vulgaris* L.) and inhibition of HepG2 cell proliferation. *J. Agric. Food Chem.* 62 1324–1331. 10.1021/jf404648u 24467616

[B16] Li Z. Zhang D. Xiong X. Yan B. Xie W. Sheen J. (2017). A potent Cas9-derived gene activator for plant and mammalian cells. *Nat. Plants* 3 930–936. 10.1038/s41477-017-0046-0 29158545 PMC5894343

[B17] Liu W. Stewart C. N. Jr. (2016). Plant synthetic promoters and transcription factors. *Curr. Opin. Biotechnol.* 37 36–44. 10.1016/j.copbio.2015.10.001 26524248

[B18] Lopez-Nieves S. Yang Y. Timoneda A. Wang M. Feng T. Smith S. A. (2018). Relaxation of tyrosine pathway regulation underlies the evolution of betalain pigmentation in Caryophyllales. *New Phytol.* 217 896–908. 10.1111/nph.14822 28990194

[B19] Marillonnet S. Grutzner R. (2020). Synthetic DNA assembly using golden gate cloning and the hierarchical modular cloning pipeline. *Curr. Protoc. Mol. Biol.* 130:e115. 10.1002/cpmb.115 32159931

[B20] Owen C. Patron N. J. Huang A. Osbourn A. (2017). Harnessing plant metabolic diversity. *Curr. Opin. Chem. Biol.* 40 24–30. 10.1016/j.cbpa.2017.04.015 28527344 PMC5693780

[B21] Polturak G. Breitel D. Grossman N. Sarrion-Perdigones A. Weithorn E. Pliner M. (2016). Elucidation of the first committed step in betalain biosynthesis enables the heterologous engineering of betalain pigments in plants. *New Phytol.* 210 269–283. 10.1111/nph.13796 26683006

[B22] Polturak G. Grossman N. Vela-Corcia D. Dong Y. Nudel A. Pliner M. (2017). Engineered gray mold resistance, antioxidant capacity, and pigmentation in betalain-producing crops and ornamentals. *Proc. Natl. Acad. Sci. U.S.A.* 114 9062–9067. 10.1073/pnas.1707176114 28760998 PMC5576821

[B23] Sasaki N. Adachi T. Koda T. Ozeki Y. (2004). Detection of UDP-glucose:cyclo-DOPA 5-O-glucosyltransferase activity in four o’clocks (*Mirabilis jalapa* L.). *FEBS Lett.* 568 159–162. 10.1016/j.febslet.2004.04.097 15196939

[B24] Sasaki N. Wada K. Koda T. Kasahara K. Adachi T. Ozeki Y. (2005). Isolation and characterization of cDNAs encoding an enzyme with glucosyltransferase activity for cyclo-DOPA from four o’clocks and feather cockscombs. *Plant Cell Physiol.* 46 666–670. 10.1093/pcp/pci064 15695438

[B25] Schornack S. Peter K. Bonas U. Lahaye T. (2005). Expression levels of avrBs3-like genes affect recognition specificity in tomato Bs4- but not in pepper Bs3-mediated perception. *Mol. Plant Microb. Interact.* 18 1215–1225. 10.1094/mpmi-18-1215 16353556

[B26] Schubert R. Dobritzsch S. Gruber C. Hause G. Athmer B. Schreiber T. (2019). Tomato MYB21 acts in ovules to mediate jasmonate-regulated fertility. *Plant Cell.* 31 1043–1062. 10.1105/tpc.18.00978 30894458 PMC6533027

[B27] Selma S. Bernabe-Orts J. M. Vazquez-Vilar M. Diego-Martin B. Ajenjo M. Garcia-Carpintero V. (2019). Strong gene activation in plants with genome-wide specificity using a new orthogonal CRISPR/Cas9-based programmable transcriptional activator. *Plant Biotechnol. J.* 17 1703–1705. 10.1111/pbi.13138 31034138 PMC6686126

[B28] Skalicky M. Kubes J. Shokoofeh H. Tahjib-Ul-Arif M. Vachova P. Hejnak V. (2020). Betacyanins and betaxanthins in cultivated varieties of *Beta vulgaris* L. compared to weed beets. *Molecules* 25:5395. 10.3390/molecules25225395 33218115 PMC7698878

[B29] Staniek A. Bouwmeester H. Fraser P. D. Kayser O. Martens S. Tissier A. (2013). Natural products - modifying metabolite pathways in plants. *Biotechnol. J.* 8 1159–1171. 10.1002/biot.201300224 24092673

[B30] Stintzing F. C. Schieber A. Carle R. (2003). Evaluation of colour properties and chemical quality parameters of cactus juices. *Eur. Food Res. Technol.* 216 303–311. 10.1007/s00217-002-0657-0

[B31] Sunnadeniya R. Bean A. Brown M. Akhavan N. Hatlestad G. Gonzalez A. (2016). Tyrosine hydroxylation in betalain pigment biosynthesis is performed by cytochrome P450 Enzymes in Beets (*Beta vulgaris*). *PLoS One* 11:e0149417. 10.1371/journal.pone.0149417 PMC4758722 26890886

[B32] Thieme F. Engler C. Kandzia R. Marillonnet S. (2011). Quick and clean cloning: a ligation-independent cloning strategy for selective cloning of specific PCR products from non-specific mixes. *PLoS One* 6:e20556. 10.1371/journal.pone.0020556 21655102 PMC3107216

[B33] Timoneda A. Sheehan H. Feng T. Lopez-Nieves S. Maeda H. A. Brockington S. (2018). Redirecting Primary Metabolism to Boost Production of Tyrosine-Derived Specialised Metabolites in Planta. *Sci. Rep.* 8:17256. 10.1038/s41598-018-33742-y PMC6250739 30467357

[B34] Tsuwamoto R. Fukuoka H. Takahata Y. (2008). GASSHO1 and GASSHO2 encoding a putative leucine-rich repeat transmembrane-type receptor kinase are essential for the normal development of the epidermal surface in *Arabidopsis* embryos. *Plant J. Cell Mol. Biol.* 54 30–42. 10.1111/j.1365-313x.2007.03395.x 18088309

[B35] Vogt T. Grimm R. Strack D. (1999). Cloning and expression of a cDNA encoding betanidin 5-O-glucosyltransferase, a betanidin- and flavonoid-specific enzyme with high homology to inducible glucosyltransferases from the Solanaceae. *Plant J. Cell Mol. Biol.* 19 509–519. 10.1046/j.1365-313x.1999.00540.x 10504573

[B36] Vogt T. Zimmermann E. Grimm R. Meyer M. Strack D. (1997). Are the characteristics of betanidin glucosyltransferases from cell-suspension cultures of *Dorotheanthus bellidiformis* indicative of their phylogenetic relationship with flavonoid glucosyltransferases? *Planta* 203 349–361. 10.1007/s004250050201 9431682

[B37] Weber E. Gruetzner R. Werner S. Engler C. Marillonnet S. (2011). Assembly of designer TAL effectors by Golden Gate cloning. *PLoS One* 6:e19722. 10.1371/journal.pone.0019722 21625552 PMC3098256

