# Supplementary material for: Engineering Betalain Biosynthesis in Tomato for High Level Betanin Production in Fruits
Source: Front Plant Sci. 2021 Jun 9;12:682443. doi: 10.3389/fpls.2021.682443 (PMC8220147; doi:10.3389/fpls.2021.682443)
Supplement: Supplementary Figure 1 — Sequence of the level 0 modules for BvCYP76AD1, BvDODA1, Db5GT, BvADHα, dTAL2, and STAP2. [file Data_Sheet_1.docx]

Supplementary information for:

Engineering betalain biosynthesis in tomato for betanin production in fruits.

Ramona Grützner^1^, Ramona Schubert^1^, Claudia Horn^1^, Changqing Yang^1^, Thomas Vogt^1^, Sylvestre Marillonnet^1*^

^1^Leibniz Institute of Plant Biochemistry, Department of Cell and Metabolic Biology, Halle, Germany

List of figures:

**Supplementary Figure 1:** Sequence of the level 0 modules for BvCYP76AD1, BvDODA1, Db5GT, BvADHα, dTAL2 and STAP2.

**Supplementary Figure 2:** List of parts and vectors for cloning level 1 constructs for transient expression and level 2 constructs for tomato stable transformation.

**Supplementary Figure 3:** List of vectors and parts deposited at Addgene

**Supplementary Figure 4:** Expression of BvCYP76AD1 and BvDODA1 in *Nicotiana benthamiana* leaves results in gomphrenin biosynthesis.

**Supplementary Figure 5:** Expression of BvCYP76AD1 and BvDODA1 in *Nicotiana benthamiana* leaves results in low amount of betaxanthins.

**Supplementary Figure 6:** Quantification of betanin production by transient expression in *Nicotiana benthamiana* leaf with and without ADHα.

**Supplementary Figure 7:** PCR analysis of the T-DNAs of pAGM48388 transformants.

**Supplementary Figure 8:** PCR analysis of the T-DNAs of pAGM51753 transformants.

**Supplementary Figure 9:** Extracts from *N. benthamiana* leaves, red beets and tomatoes.

**Supplementary Figure 10:** Analysis of the T-DNA insertion site in pAGM51753 transformant 2.

**Supplementary Figure 11:** PCR analysis of selfed progenies of pAGM51753 pt2.

**Supplementary Figure 12:** List of backcrosses of pAGM51753 transformant 2 with tomato cultivars Moneymaker and M82.

**Supplementary Figure 13:** Fruits obtained after 3 backcrosses with M82 or Moneymaker.

**Supplementary Figure 14:** Plasmid stability of high copy plasmids in *E.coli* and Agrobacterium.

**Supplementary Figure 15:** Plasmid stability of low copy plasmids in *E.coli* and Agrobacterium.

**Supplementary Figure 1: Sequence of the level 0 modules for BvCYP76AD1, BvDODA1, Db5GT, BvADHα, dTAL2 and STAP2.** Flanking BsaI sites are shown in red. The start and stop codons are shown in bold characters.

pAGM1021 (level 0 module for BvCYP76AD1)

ggtctc a a**atg** gatcatgcaacattagcaatgatactagccatttggttcatttcttttcatttcataaaattactttttagccaacaaactaccaaacttcttcctcctggtccaaaaccattgccaataattggtaacatcttagaagttggtaaaaaaccccatcgttcatttgctaatcttgctaaaattcacggccctttaatatcgttacgtctaggaagtgtaacaactattgttgtatcatcagcagatgtagctaaagaaatgttcttaaaaaaagaccaccctctttctaaccgtactattcctaattctgtcacggccggtgaccaccataaactcaccatgtcgtggttgcccgtttcgccgaaatggcggaattttcgtaagattacagccgtccatttgctttctcctcaaagacttgatgcttgccaaacctttcgccatgccaaggtgcaacagctttatgaatatgtacaagaatgtgcacaaaaaggccaagctgttgatattggcaaagctgcatttactacctcccttaatttgttatctaaactattcttttcagtggaattagcccaccataaatcacacacttctcaagagtttaaggaactaatatggaacattatggaagatattgggaaacctaattatgctgattattttcctattttaggctgtgttgatccatcaggtattcgtcgaagattagcatgtagttttgacaagttgattgcagtttttcagggtataatatgtgaaaggcttgcgcctgattcttcaactacaacaacaacgacgactgatgatgtgctagacgttcttcttcagctgttcaaacaaaatgagctcactatgggcgagattaaccatttgctcgtcgacatttttgatgctggaacagacactacatcaagtacttttgagtgggtcatgacagagttaattaggaatcctgaaatgatggaaaaggctcaagaagaaattaagcaagtattgggcaaagataaacaaattcaggaatcagacattattaacctaccttacttacaagccattatcaaagaaactttgcgactacatccaccaactgtatttcttttgcctcgtaaagccgacactgatgttgaactatatggttatattgtgcctaaagatgcacaaatacttgttaacttatgggctattggaagagatcctaatgcatggcaaaatgctgatattttttcgcccgaaagatttatagggtgtgaaattgatgtcaaaggaagagattttggactcttaccttttggagccggaagaaggatatgtcctgggatgaatttggccattagaatgttaactttgatgctagctactttacttcaattcttcaattggaagcttgaaggagacataagtccaaaagacttagacatggatgagaaatttgggattgcgttacaaaagacaaagcctttaaaacttattccaatacctaggtat **tga** gctt t gagacc

pAGM862 (Level 0 module for BvDODA1)

ggtctc a a**atg** aaaatgatgaatggtgaagatgctaatgatcaaatgatcaaagaaagcttcttcataacacatggaaacccaatattaacagtagaagatacacatccattaagacctttctttgaaacttggagagagaaaatcttttctaagaaacctaaggcaattcttattatttctggtcattgggaaactgttaaacctactgttaatgctgtccatatcaatgatactatccatgattttgatgactatcctgctgctatgtaccagttcaagtatccagctcctggggaaccagaattggcaagaaaagtagaggaaattctgaaaaaatcgggtttcgaaacggcggaaactgatcaaaaacgtgggcttgatcatggtgcatgggtacctctaatgctaatgtatcctgaggctgatataccagtatgtcagctctcagttcagccgcatttagatggaacataccattacaacttaggacgagcattggctcccttgaaaaacgacggcgtattaatcattggttcaggaagtgcaactcaccctttggatgaaactcctcattattttgatggagttgcaccttgggcagctgcctttgattcatggcttcgtaaagctctcattaatggaaggtttgaagaagtgaatatatatgaaagcaaagcaccaaattggaaattagcacatcctttcccagaacatttttatccattgcatgttgttcttggcgctgctggtgaaaaatggaaggcagagcttattcatagcagttgggatcatggcaccttatgtcatggctcctacaagttcacttcagcc **tag** gctt t gagacc

pAGM921 (Level 0 module for Db5GT)

ggtctc a a**atg** ggtacacactcaacagcaccagatctccatgtagttttcttcccattcttggctcatggccacatgatcccgtccctagacatcgcgaagctctttgcagctcgcggtgtcaaaacaaccatcatcaccacccctcttaacgcatccatgttcaccaaagcaatagaaaaaaccagaaaaaacacagaaactcagatggaaattgaagttttcagcttcccgtctgaagaagctggtttgccccttggatgtgagaatttggagcaagccatggctattggtgcaaataatgaattctttaatgctgcgaatttgctgaaagagcagctggagaattttctagtgaaaactcgaccaaattgccttgtggccgatatgttcttcacctgggcagctgattccacagccaaattcaacatccctaccctagtttttcatggatttagctttttcgcccaatgtgccaaagaggttatgtggaggtataagccttataaggccgtgtcatccgacacagaggtattttctcttccatttcttccccatgaggtgaagatgaccagattgcaggttccagagagtatgaggaaaggtgaggaaactcacttcactaagagaacagaacgcatcagggaattggaacgcaagagttatggggtgattgttaacagcttttacgagttggagcccgattatgcagactttcttaggaaagaattggggagaagggcatggcatataggccctgtttcactatgcaataggagcatcgaggataaagctcaacgagggagacaaacttcaatcgatgaagatgaatgcttgaaatggcttaactccaaaaaacccgattcagttatctatatctgtttcggaagcacaggacacctcatagcccctcagttacacgagattgcaacggctctagaagcttccggacaagactttatctgggcggtgaggggtgatcatggtcaaggaaatagcgaagagtggttgccaccgggatacgaacatagattgcaagggaaaggcttgataatacgagggtgggctccacaggtgttgattttggagcacgaagctacaggaggatttctgactcactgtgggtggaactcggcacttgaagggatctctgctggtgtgccaatggttacatggcctacttttgccgagcaattccataacgagcagttgttgacacagattttgaaggtcggggtcgctgttggatctaagaagtggaccctaaagccttctatcgaagatgtgataaaagcggaagatatagagaaggcagttagagaagttatggtgggagaggaaggtgaagaaaggagaaggagggcaaagaagttgaaggaaatggcatggagggctattgaagaaggtggatcatcctactcggatttaagtgcactgattgaggaattgaagggttatcatacttcagagaaagag **tag** gctt t gagacc

pAGM49981 (Level 0 module for BvADHα)

ggtctc a a**atg** atttcactctcttcttttcatccttcctccaccaccgccaccgccaccgccgccgccgccaccacccacccacctcaacaatgtcccgctttttcctctcctccgtcgcatctctcgcttcctttacgccaccctcgccaacaccttgtagttcggtgcggtggaggtggttcggcctccgaatcggtatttaaccgtgatagtgctgctactcgtgtttctaatgatcatcttgacgttagtaaaagagatgttaagcttaagattgctattattgggtttggtaactttggccagtttttggctaagacaatggctaagcaaggtcatagagtgttggcttactcacgctcggactactcccgcgctgctaaggagatcggcgtcgagtattttactgacgccgatgacctctgcgaggagcaccctgaggttattctgttgtgcacatccatcctctcaacggagaaggtcctccgatcactccccctccaccggctccgtcgttcaaccctctttgcggatgttctctcggtcaaggaatttcctcgatcgctcttccttcaactacttcctaaggactttgatatcctatgcacccaccctatgtttggcccagactcgggcaaagacgggtggggtggactaccttttgtgttcgataaagttagagtcggatcagatcagagtcggacatctcgtgctgaggcattcctagacgtgtttaggaatgccgggtgtaggatggtggaaatgagttgtgttgatcatgacaagcatgcagccggatctcaatttattacacatatgatgggacgagttttggagaaattggccttggaaaatacaccaattaatacaaaagggtacgaaagtttgttaaatttggtggataatactgcaagggatagttttgagttgttttacgggttgtttttgtacaataaaaatgcaatggagcaattggatagaatggattgggctttcgagatggtaaaaaagcaactttcgggatatttgcatgatcttgttagaaaacaattgatgttggagggtaataatgatcaagctgaggttacttttgacaaaccattgatgcttccttctcctactattaatcctccacaaatagttccctctgctgatatggctgagaagaagcatgatttagtggtggttaatggtactaga **tag** gctt t gagacc

pICH73103 (dTAL2 level 0 module)

ggtctc a a**atg** gatccgattcgttctcggacaccttctcccgctcgagagctccttcctggaccacaaccagatggagtgcaaccaacagctgataggggtgtatctcctccagccggtggacctcttgacggcttacctgcaaggcgcacaatgtcccgtaccagattgcccagtccaccagcaccaagtccagcgttttcagcgggcagcttctctgacctcctgagacagtttgatccctctttgttcaacacctcactgtttgactcacttccgccttttggggctcaccatactgaagccgctactggtgagtgggatgaggtgcagtcaggcttaagagcagctgatgccccaccacctacgatgagggtggcagtaactgctgctaggccaccgagagctaaaccagctccaagacgcagagcagcacaaccttccgatgcatctcctgctgctcaagtcgatcttcgcactttagggtatagccagcaacaacaggagaagatcaaacctaaggtccgaagtacagttgcgcaacaccatgaagcccttgttggtcatgggttcactcatgcccacatagttgcactatcccaacatcctgctgctcttggaactgttgcggtgaagtaccaggacatgattgctgctttacctgaagcaacacacgaggcaatagtcggtgttggcaaacagtggtctggcgctagggctctagaagccctccttacagttgcaggagaattgcggggacctcccttgcagctcgataccggacaattgctgaagattgccaaacgtggtggtgttacggcagtagaagcagtgcatgcttggaggaatgctctaactggagcacccttgaatctcacgccagaacaagtggtcgctatcgcctcccatgatggtggaaaacaagcactagaaactgtccaaagattattgcctgttctttgtcaggcacacggacttaccccacaacaagtcgttgctatagcctcccatgatggagggaagcaagcgttagaaacagtgcagcggctactccctgtattatgccaggctcatggtctaactccacaacaagtggtggctatagcctcacacgatggtggtaaacaggcacttgaaaccgtccaaagactcctgccggtcctctgccaggcacacggcctcacccccgaacaagtggtggctattgcttcgcatgatggaggtaagcaggctttagagacagtccagagactactacccgttctatgccaggcccatggtttgaccccggaacaggttgttgctattgcgtcaaataagggcggcaagcaagcgttggaaaccgttcaagcattactccctgttctctgtcaagcacatgggctaacgcccgagcaggttgttgcaattgcatcacatgatggaggaaagcaggccttagaaacggtacaggcacttttaccagtcctttgccaagcacacgggcttacacccgaacaagtggtcgctattgcaagtaatataggtggaaaacaagcactggaaaccgtgcaggcgcttttgccggtattatgccaagctcacggcctaactcctgaacaggtggttgcgattgcctcaaatggtgggggtaaacaggcactggagactgtgcagcggcttttgcctgttttgtgtcaagctcatggattgacaccagagcaggtggtcgctatagctagtaacattggaggtaaacaagcgcttgaaaccgtgcaacgtctgctgccagttctatgtcaagctcatgggttgaccccacaacaggttgtagcgatcgcttccaataaaggaggaaagcaagctctagaaacggtgcagaggctcctcccggttctttgtcaggcgcatggattgaccccggagcaggtggtcgcaatcgccagtcatgatggaggtaagcaggccttggaaaccgttcaggcgttactcccggttctatgccaggcgcatggcctgacccctgaacaggttgtggcgatagccagtaacggcgggggaaagcaggcacttgaaaccgtacaacgactcctcccagtcctttgtcaagcccacggattgactccagaacaagtagttgctatagcttcgaataagggaggaaagcaggcccttgaaacagttcagcgtcttttgccagtgttgtgtcaagcacacggattgactcctgaacaggttgtcgccattgcatctaatatcggtggtaagcaagctctcgaaaccgtacagcgactcttgcctgttctatgccaagcgcatggcttgacgccggaacaggtggtagccatagcaagcaacataggtggcaaacaagctcttgaaacagttcaaaggttgttacctgtgctttgccaagcccacggtttgacccctcaacaggtggttgctatagcatcacatgatgggggacggcctgctcttgagacagtgcagcgcctgttgcccgtgttgtgtcaagcgcatggcttaacaccggaacaggtcgtggcaattgcgtcaaatattggcggcaaacaagcgctggaaaccgttcagcgactcttgcctgttctgtgccaagctcacggtctgacgccccaacaggttgttgccattgcttcaaatggaggagggaggccagcccttgagtcgattgtcgcacagctatctcggcccgaccctgctttagccgctctgacaaatgatcatcttgtggctctcgcctgcttaggaggtcgcccagctttagacgcagtaaaaaagggtctacctcatgctccggccttaatcaagaggacgaatcgtagaatcccagaacgaacgagccatcgcgtagccgatcacgctcaagttgttagggttttaggtttttttcagtgtcattcacatccggcacaagctttcgatgatgccatgacccagtttggtatgtcaaggcatggattactgcaacttttcagaagagtaggagtgacagagctcgaagccagaagcggaactctgccacccgctagccaaagatgggataggatattgcaggcgagtggaatgaagcgcgcgaaaccatctccaacaagcactcaaaccccggatcaagcgagtttgcacgctttcgcagattctctcgaacgagatttggatgccccttctccaatgcacgaaggtgatcaaactagggcgagtagcaggaagaggtctaggagtgatcgtgcagttacgggcccctcagcacaacagtcttttgaggtcagggtgccagaacaaagggacgctttacatctcccattgtcttggcgtgtaaaaaggccgcgaactagtattggagggggattaccggacccagggacccccactgctgctgatctagctgcttctagtacggtaatgcgcgagcaagacgaggatccatttgctggggcagctgatgacttccccgcattcaacgaagaagaattagcatggttgatggagttactgccacag **taa** gctt t gagacc

pICH71037 (STAP2 level 0 module)

ggtctc a ggag tttttttgtacttttgttgtccccgcatagctgaacatctatataattaaaaaatagaccagataacaacagaaataccagaacataat a**atg** t gagacc

pAGM24951 (E8 promoter level 0 module)

ggtctc a ggag ttttgtggaagtgagagagtccaattttgataagaaaagagtcagaaaacgtaatattttaaaagtctaaatctttctacaaataagagcaaatttatttattttttaatccaataaatattaatggaggacaaattcaattcacttggttgtaaaataaacttaaaccaataaccaaagaactaataaatcctgaagtggaattattaaggataaatgtacatagacaatgaagaaataataggttcgatgaattaataataattaaggatgttacaatcatcatgtgccaagtatatacacaatattctatgggatttataatttcgttacttcacttaacttttgcgtaaataaaacgaattatctgatattttataataaaacagttaattaagaaccatcatttttaacaacatagatatattatttctaatagtttaatgatacttttaaatcttttaaattttatgtttctttagaaaataaaaattcaaaaaattaaatatatttacaaaaactacaatcaaacacaacttcatatattaaaagcaaaatatattttgaaaatttcaagtgtcctaacaaataagacaagaggaaaatgtacgatgagagacataaagagaactaataattgaggagtcctataatatataataaagtttattagtaaacttaattattaaggactcctaaaatatatgataggagaaaatgaatggtgagagatattggaaaacttaataattaaggattttaaaatatatggtaaaagataggcaaagtatccattatccccttttaacttgaagtctactaggcgcatgtgaaagttgattttttgtcacgtcatatagctataacgtaaaaaaagaaagtaaaatttttaattttttttaatatatgacatattttaaacgaaatataggacaaaatgtaaatgaatagtaaaggaaacaaagattaatacttactttgtaagaatttaagataaatttaaaatttaatagatcaactttacgtctagaaagacccatatctagaaggaatttcacgaaatcggcccttattcaaaaataacttttaaataatgaattttaaattttaagaaataatatccaatgaataaatgacatgtagcattttacctaaatatttcaactattttaatccaatattaatttgttttattcccaacaatagaaagtcttgtgcagacatttaatctgacttttccagtactaaatattaattttctgaagattttcgggtttagtccacaagttttagtgagaagttttgctcaaaattttaggtgagaaggtttgatatttatcttttgttaaattaatttatctaggtgactattatttatttaagtagaaattcatatcattacttttgccaacttgtagtcataataggagtaggtgtatatgatgaaggaataaacaagttcagtgaagtgattaaaataaaatataatttaggtgtacatcaaataaaaaccttaaagtttagaaaggcaccgaataattttgcatagaagatattagtaaatttataaaaataaaagaaatgtagttgtcaagttgtgttcttttttttggataaaaatagcagttggcttatgtcattcttttacaacctccatgccacttgtccaattgttgacacttaactaattagtttgattcatgtatgaatactaaataattttttaggactgactcaaatatttttatattatcatagtaatatttatctaatttttaggaccacttattactaaataataaattaactactactatattattgttgtgaaacaacaacgttttggttgttatgatgaaacgtacactatatcagtatgaaaaattcaaaacgattagtataaattatattgaaaatttgatatttttctattcttaatcagacgtattgggtttcatattttaaaaagggactaaacttagaagagaagtttgtttgaaactacttttgtctctttcttgttcccatttctctcttagatttcaaaaagtgaactactttatctctttctttgttcacattttattttattctattataaatatggcatcctcatattgagatttttagaaattattctaatcattcacagtgcaaaagaa a**atg** t gagacc

**Supplementary Figure 2: List of parts and vectors for cloning level 1 constructs for transient expression and level 2 constructs for tomato stable transformation.** A BAR cassette located just upstream the right border in some of the level 1 construct helps increase transient expression of the transcription unit present upstream. pSPM is the promoter of maize transposable element Spm. The BAR cassette was not tested for plant transformation and is used here only as buffer sequence. For the level 1 and 2 expression constructs, the name of the level 0 modules used for construction of the level 1 constructs is shown below the parts.


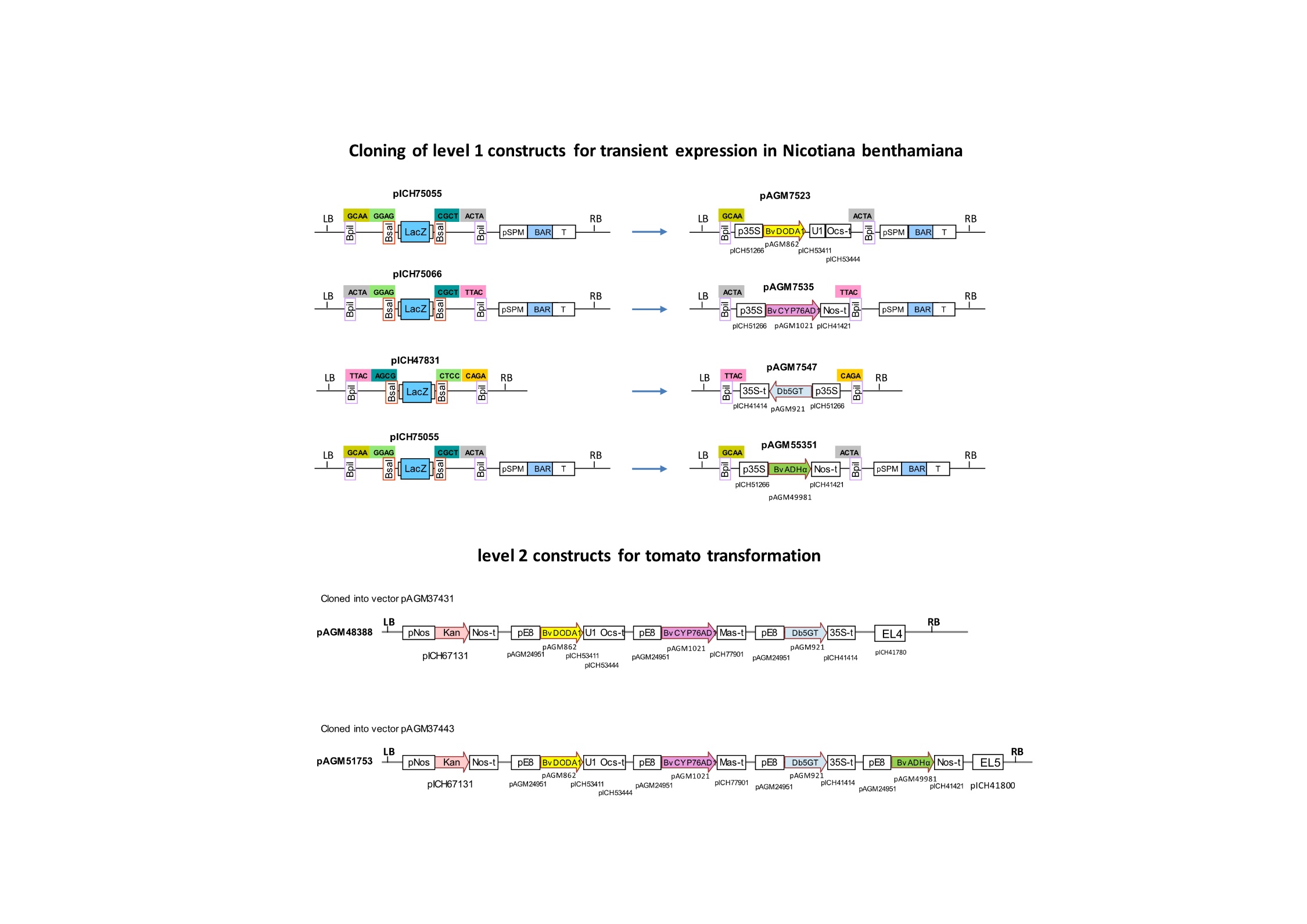


**Supplementary Figure 3: List of vectors and parts deposited at Addgene.** pAGM53151 is a cloning vector for transient expression in *Nicotiana benthamiana*. It already contains the 35S promoter and the nos terminator. Level 0 coding sequence modules can be subcloned in this vector using BsaI. Two low copy level 2 and P vectors, pAGM62624 and pAGM62636, are provided. They both have a p15a ori for low/medium copy replication in E.coli, and the Agrobacterium rhizogenes A4 ori for replication in Agrobacterium at 1 to 2 copies. SRED is a carotenoid operon for red/white selection of colonies containing an insert.

**
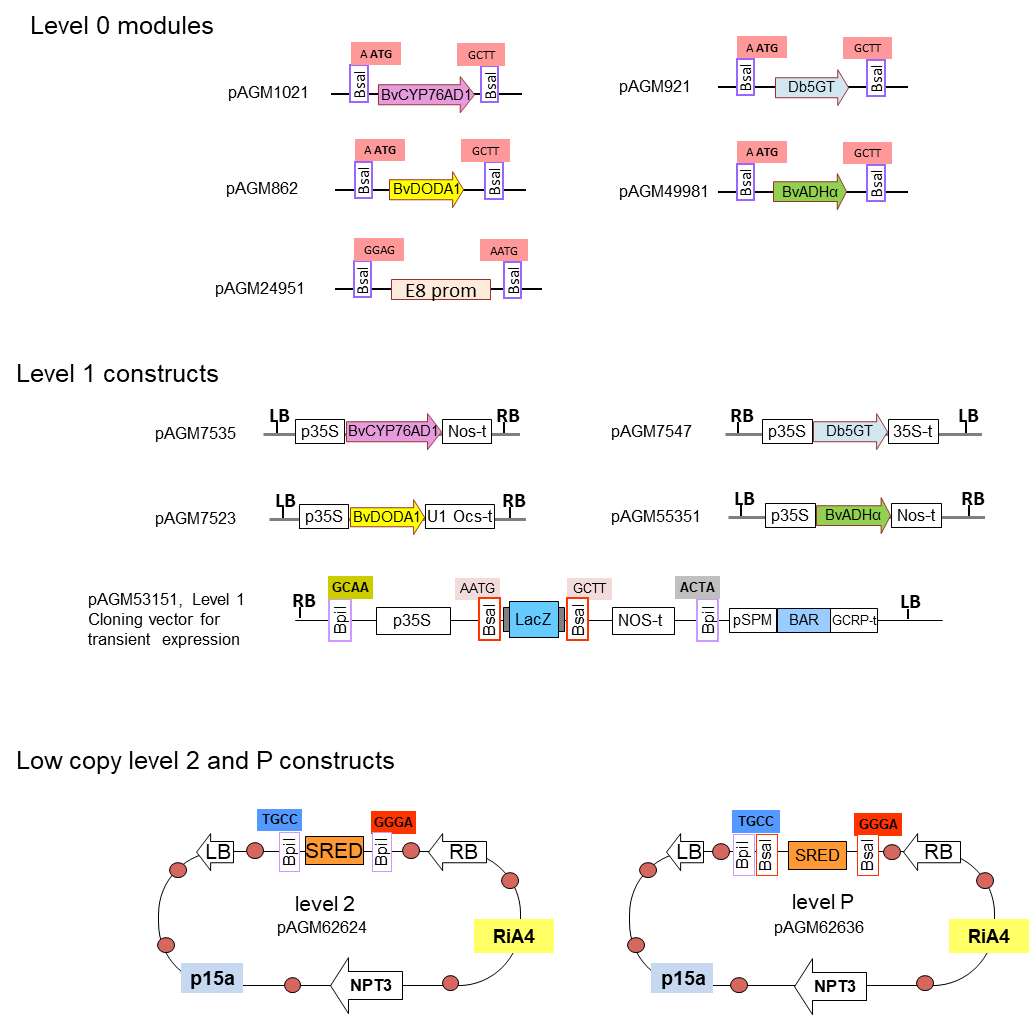
**

**Supplementary Figure 4: Expression of BvCYP76AD1 and BvDODA1 in *N. benthamiana* leaves results in gomphrenin I (betanidin-6-*O*-β-D-glucoside) biosynthesis (A) confirmed by an authentic gomphrenin I standard (B).**


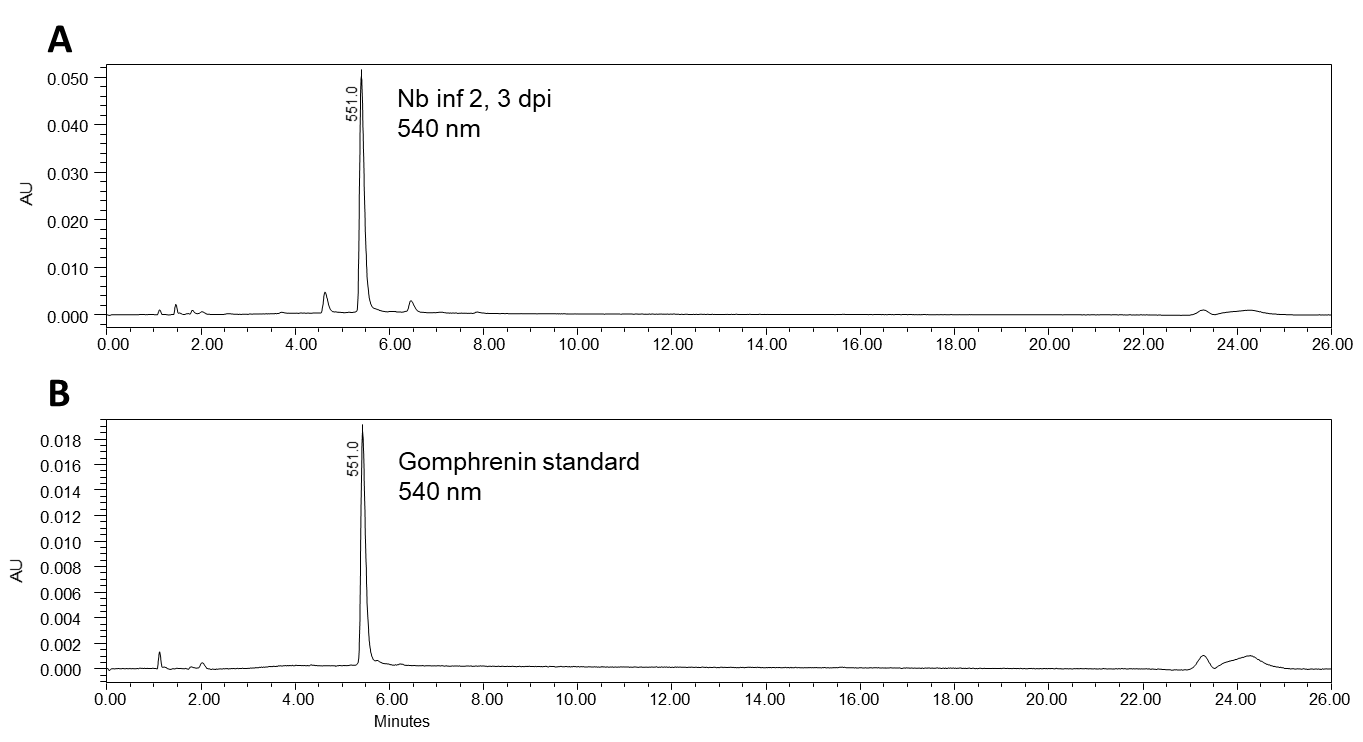


**Supplementary Figure 5: Expression of BvCYP76AD1 and BvDODA1 in *N. benthamiana* leaves results in low amount of betaxanthins**. **(A)** LC/MS analysis of an extract from red beet. Infiltration of different *N. benthamiana* leaves for different times **(B, C)** leads to different relative amount of vulgaxanthin I (peak 4), an unidentified betaxanthin (peak 6), and gomphrenin I (peak 5).

**
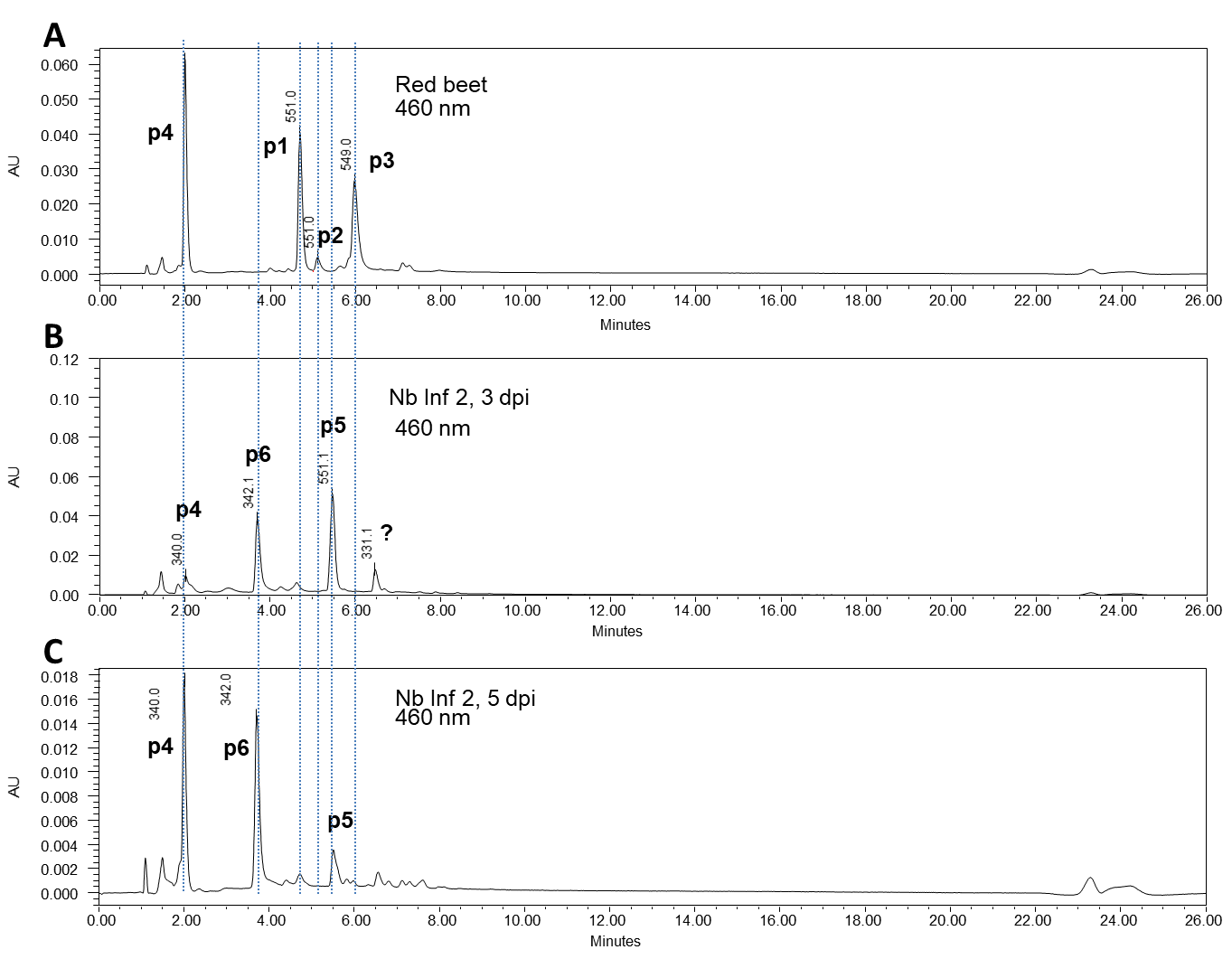
**

**Supplementary Figure 6: Quantification of betanin production by transient expression in *N. benthamiana* leaves with and without ADHα.** (A) *N. benthamiana* leaf expressing betanin biosynthesis constructs without and with ADHα. (B) The extracts shown here were prepared from 12 mg of infiltrated leaf samples homogenized in 120 µl of methanol buffer (50% methanol, 1 mM ascorbic acid, 0,5 % formic acid. (C) The extracts were then further diluted 12 fold for betanin quantification.

**
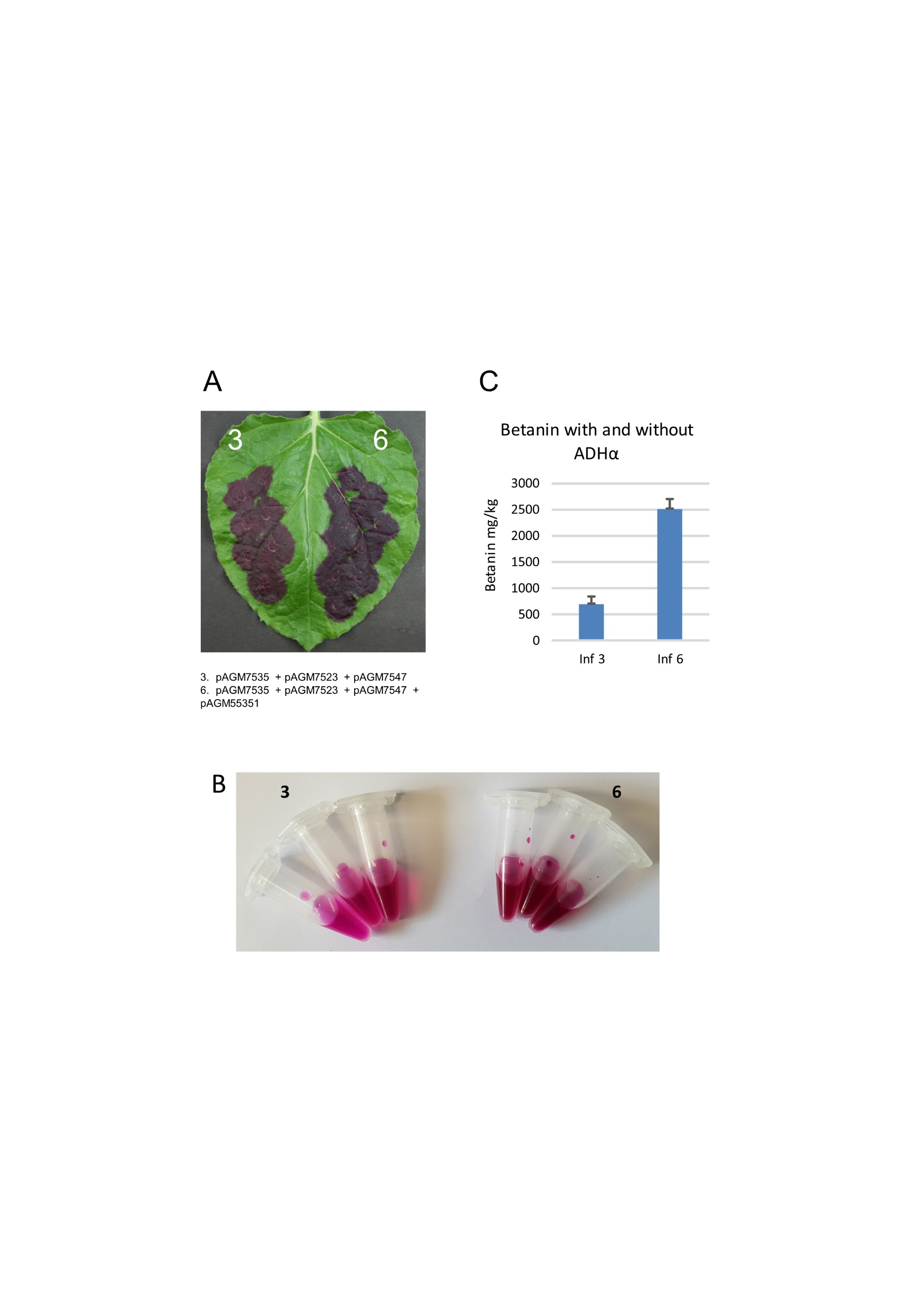
**

**Supplementary Figure 7: PCR analysis of the T-DNAs of pAGM48388 transformants. (A)** PCR analysis of the transformants. Transformants 1 to 8 were analyzed with gene-specific primer pairs kanpr1 (ATGACTGGGCACAACAGAC) kanpr2 (AAGGCGATAGAAGGCGATG), 713 bp; cyppr1 (CCAACAAACTACCAAACTTCTTCC) cyppr3 (ctagcacatcatcagtcgtcgttg), 746 bp; Dodapr1 (CTGCTATGTACCAGTTCAAGTATC) Dodapr2 (TCTGCCTTCCATTTTTCACC), 508 bp; gtpr2 (CCCTCCTTCTCCTTTCTTCAC) gtpr3 (ccgtgtcatccgacacagagg), 852 bp. M is the GeneRuler 1kb Plus DNA ladder from ThermoFisher. **(B**) Structure of the T-DNAs in transformants 1 to 8.

**
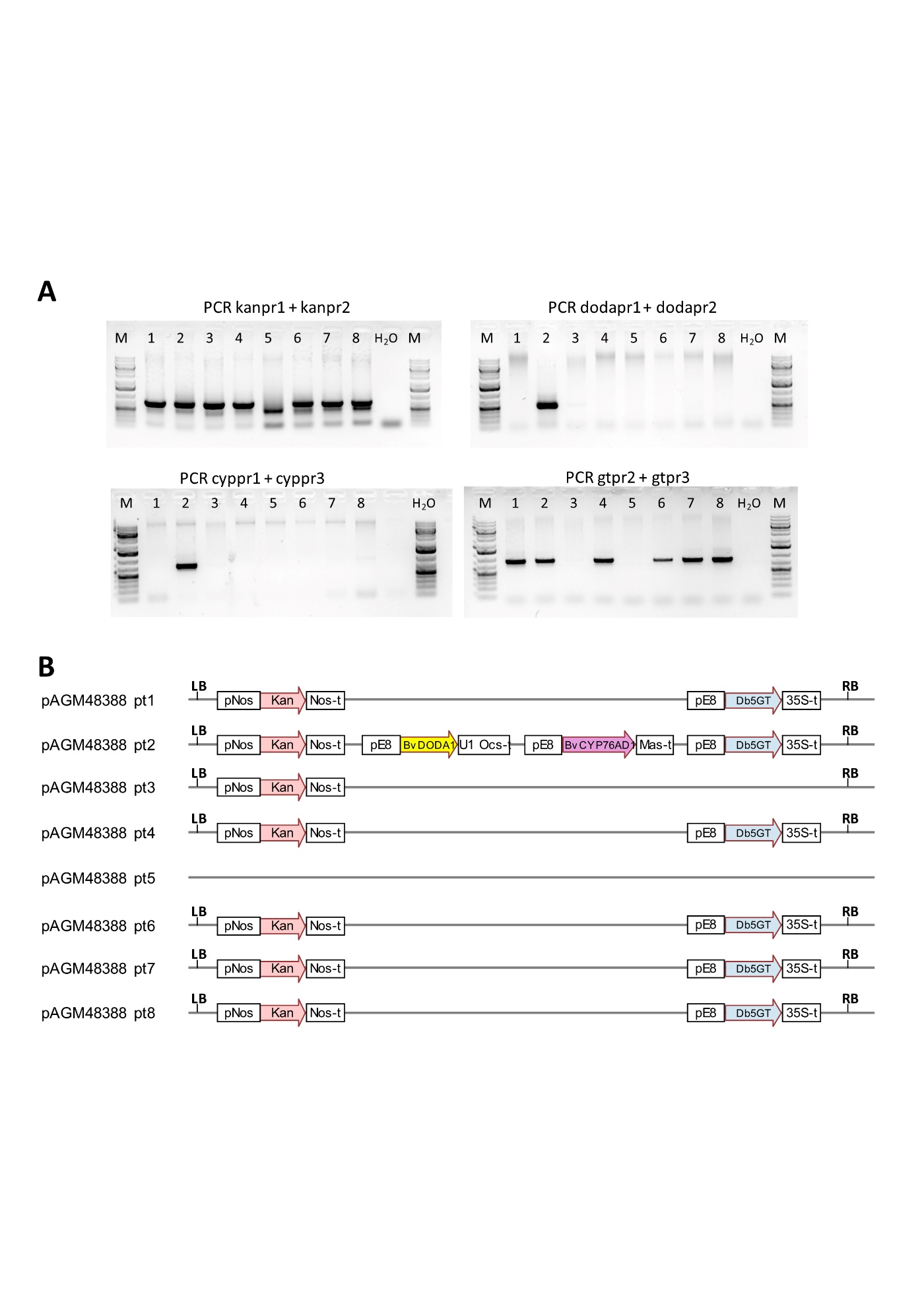
**

**Supplementary Figure 8: PCR analysis of the T-DNAs of pAGM51753 transformants. (A)** PCR analysis of the transformants. Transformants 1, 2, 3, 4 and 7 were analyzed with gene-specific primer pairs cyppr1 (CCAACAAACTACCAAACTTCTTCC) cyppr2 (CGCAAGCCTTTCACATATTATACC), 700 bp; Dodapr1 (CTGCTATGTACCAGTTCAAGTATC) Dodapr2 (TCTGCCTTCCATTTTTCACC), 508 bp; gtpr1 (TTTTCTCTTCCATTTCTTCCCC) gtpr2 (CCCTCCTTCTCCTTTCTTCAC), 829 bp; adhpr1 (ctcgccaacaccttgtag ttcg) adhpr2 (ccaaaactcgtcccatcatatgtg), 693 bp. For amplification of ADHα from transformant 4, a weak band is probably derived from some contamination from DNA. C is amplification from the control plasmid pAGM51753. M is the GeneRuler 1kb Plus DNA ladder from ThermoFisher. **(B**) Structure of the T-DNAs in transformants 1, 2, 3, 4, 7 and 8. pAGM51753 pt8 was not analyzed by PCR. The structure shown is expected, as this transformant produced betalains.

**
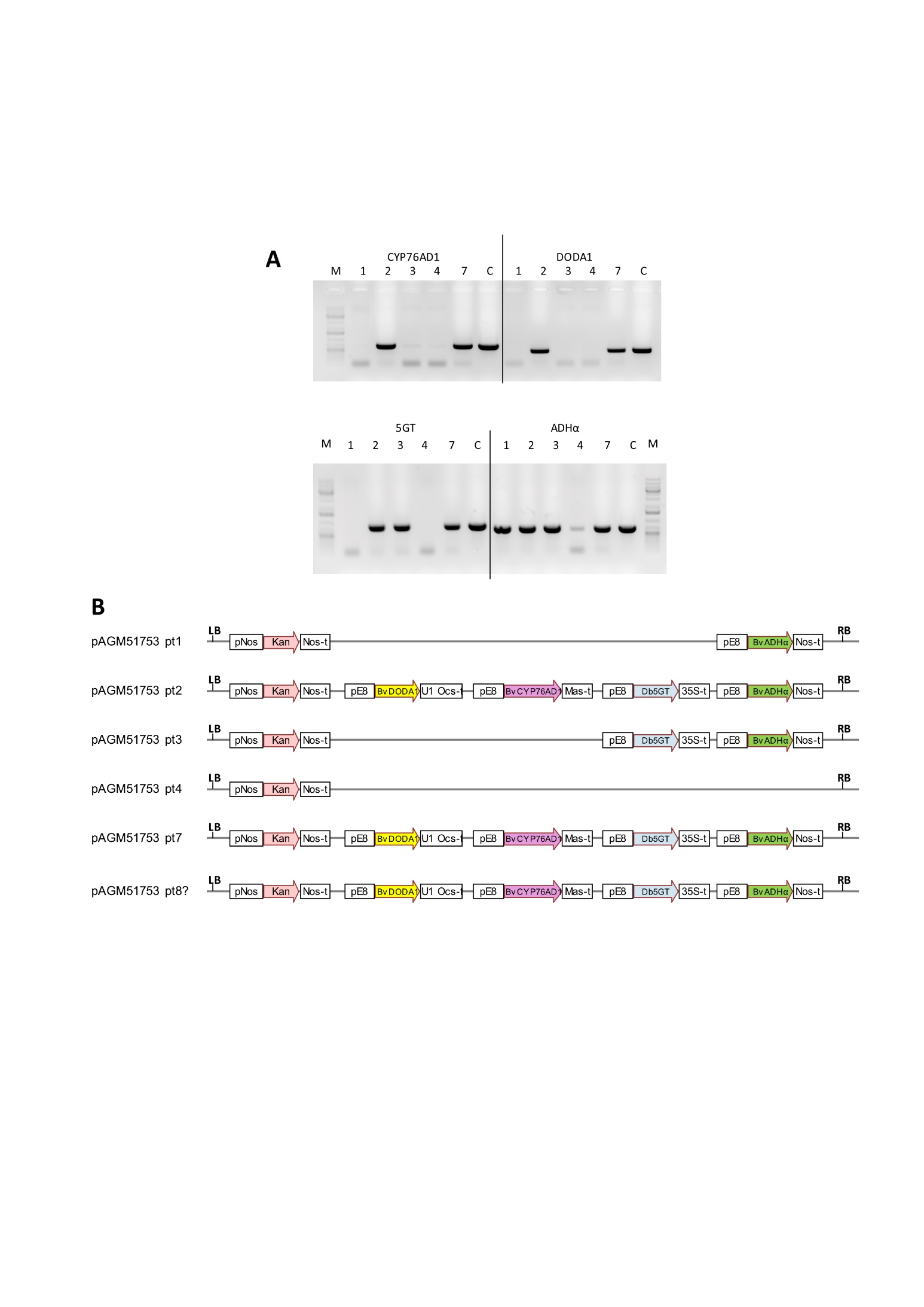
**

**Supplementary Figure 9: Extracts from *N. benthamiana* leaves, red beets and tomatoes.** The extracts shown here were prepared from 12 mg to 50 mg of tissue homogenized in methanol buffer (50% methanol, 1 mM ascorbic acid, 0,5 % formic acid) in the ratio 120 µl buffer for 12 mg tissue. The extracts were later diluted 12 fold for betanin quantification. (A) Extracts from the *N. benthamiana* leaf shown on the right, and of three red beets (one of them shown on the right). (B) Extracts from tomato crosses with Moneymaker (MM) or M82; the pictures of some fruits is shown on the right, with some fruits producing betalains, and one control fruit from the same cross lacking the T-DNA. For the cross pAGM51753 pt2 x MM, an extract from one fruit lacking betalains is shown.

**
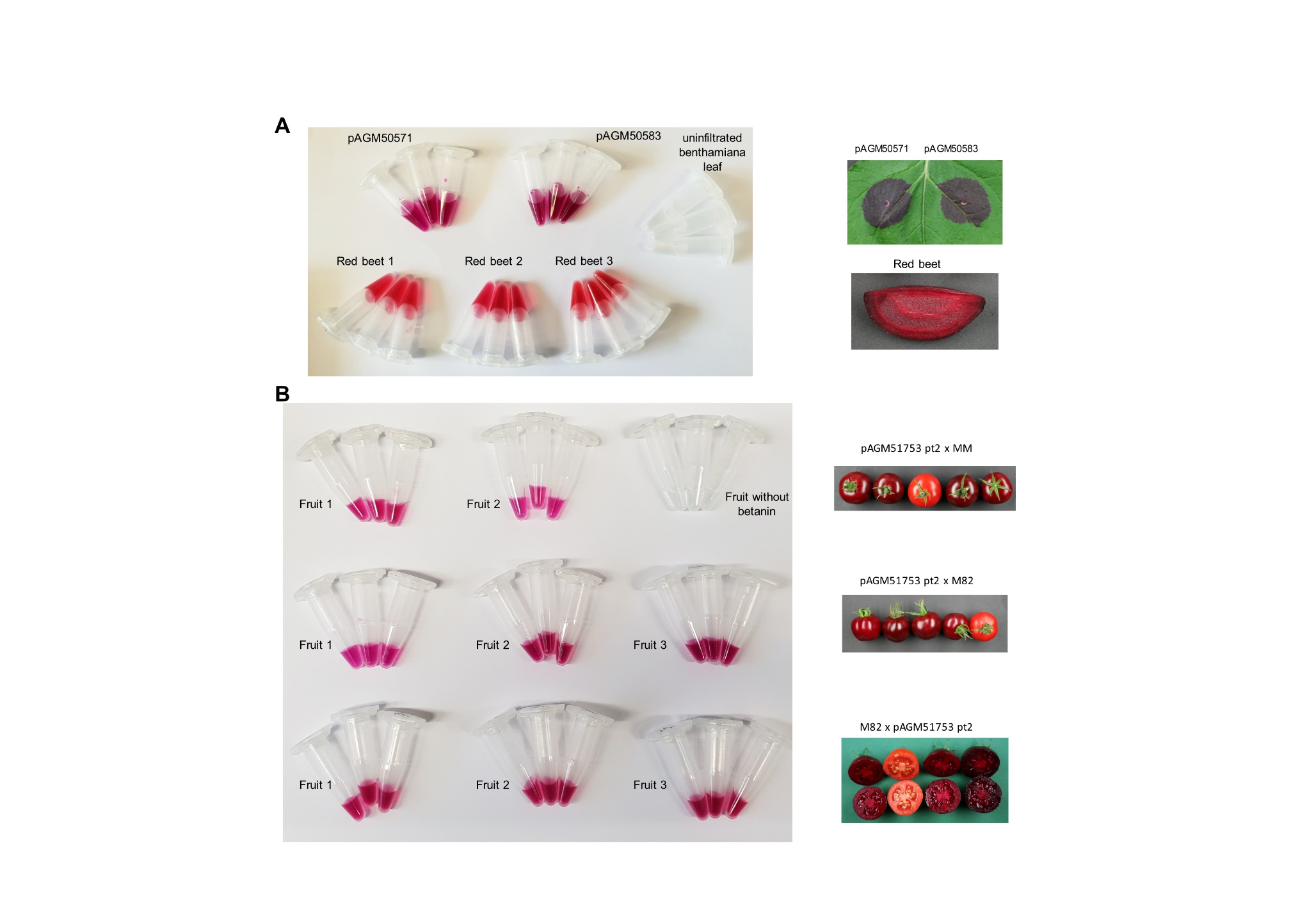
**

**Supplementary Figure 10: Structure of the T-DNA insertion site in pAGM51753 transformant 2.**

**
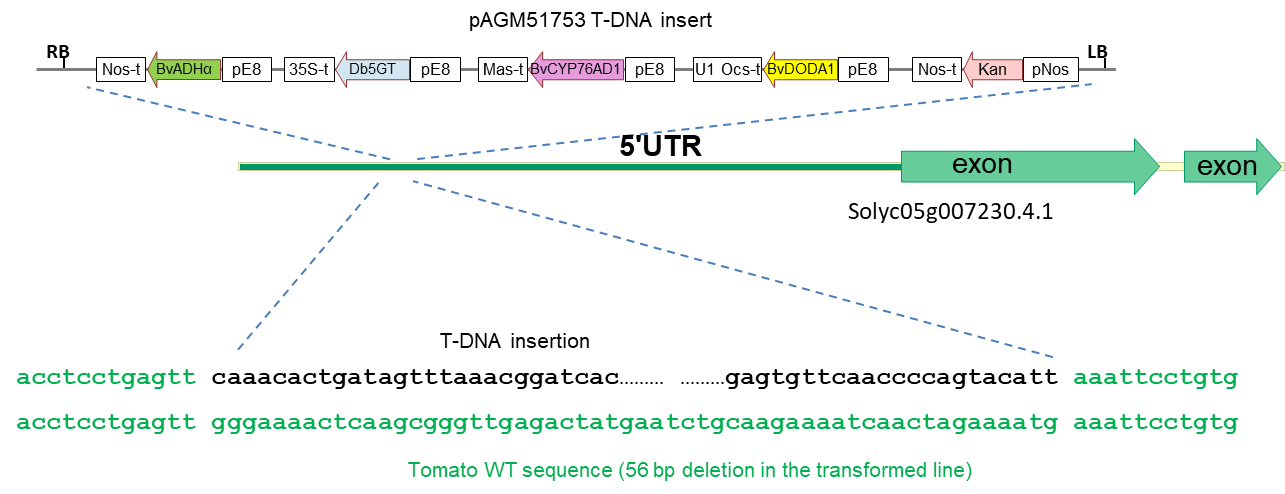
**

**Supplementary Figure 11: PCR analysis of selfed tomato progenies of pAGM51753 pt2.** (A) Structure of the T-DNA at the insertion site and position of the primers for PCR analysis. (B) PCR analysis for presence of the T-DNA in 7 self progeny plants of pAGM51752 pt2, and pictures of fruits of the corresponding plants. All plants are heterozygous expect one plant without T-DNA (plant 4) that does not produce betanin. (C) PCR analysis for presence of the T-DNA in 15 additional self progeny plants of pAGM51752 pt2 with a different set of primers.

**
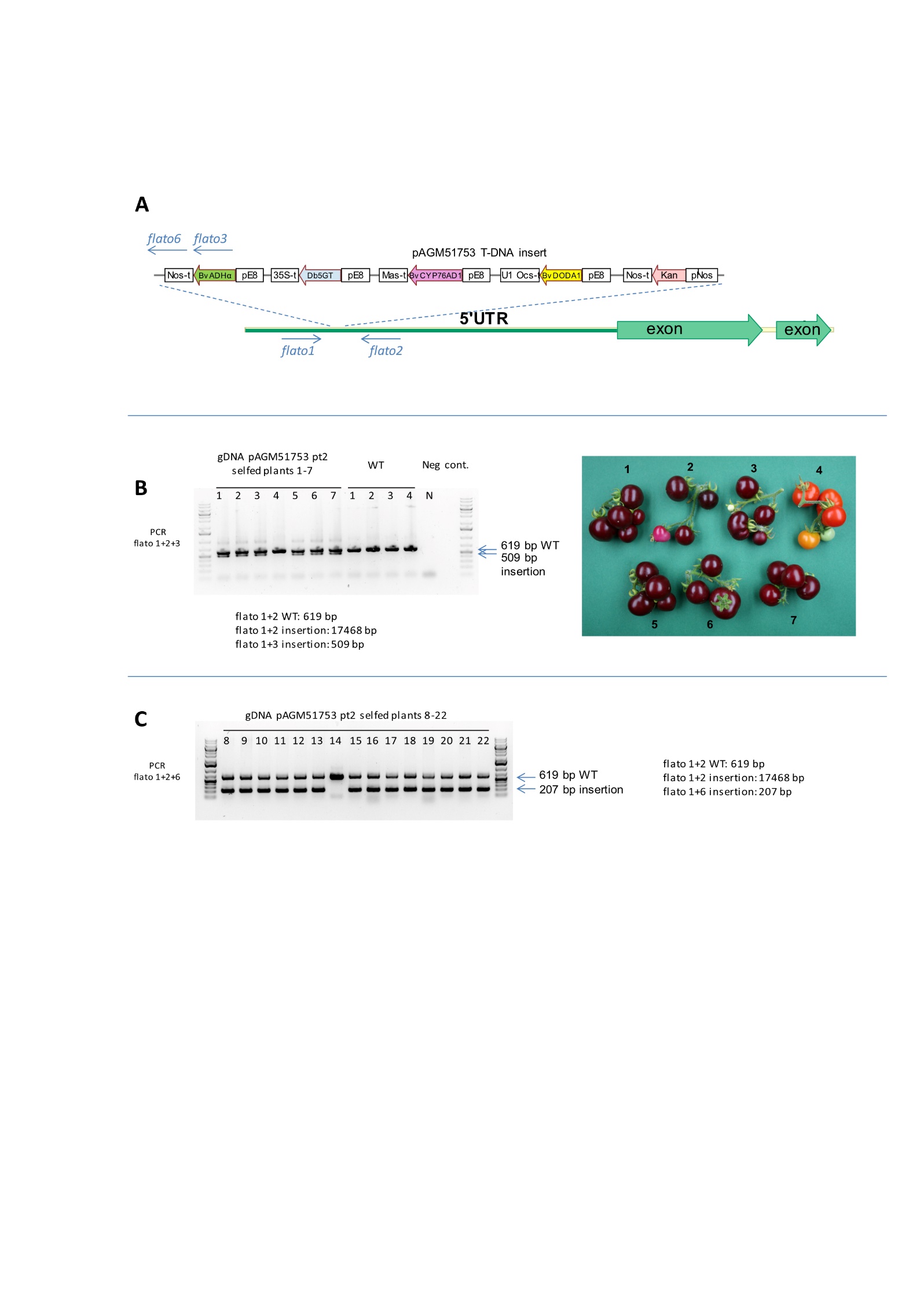
**

**Supplementary Figure 12: List of backcrosses of pAGM51753 transformant 2 with tomato cultivars Moneymaker and M82.**

**
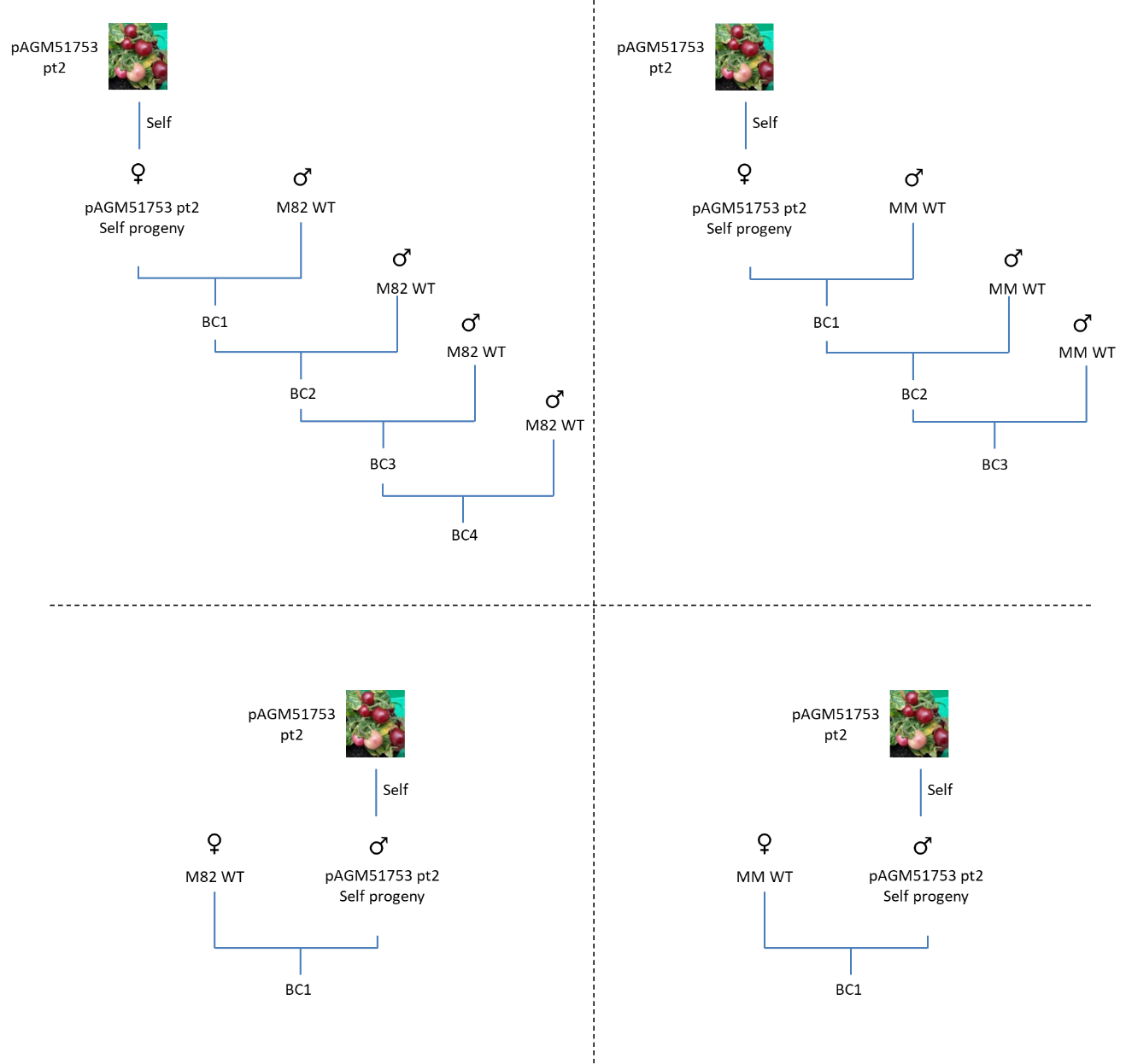
**

**Supplementary Figure 13: Fruits obtained after 3 backcrosses with M82 or Moneymaker.** (A) Fruits of the backcrosses 3 plants of pAGM51753 pt2 x M82 (top image) or pAGM51753 pt2 x Money maker (lower image). (B) The same fruits were cut in half and blotted on paper, M82 cross on top, Moneymaker cross, below.

**
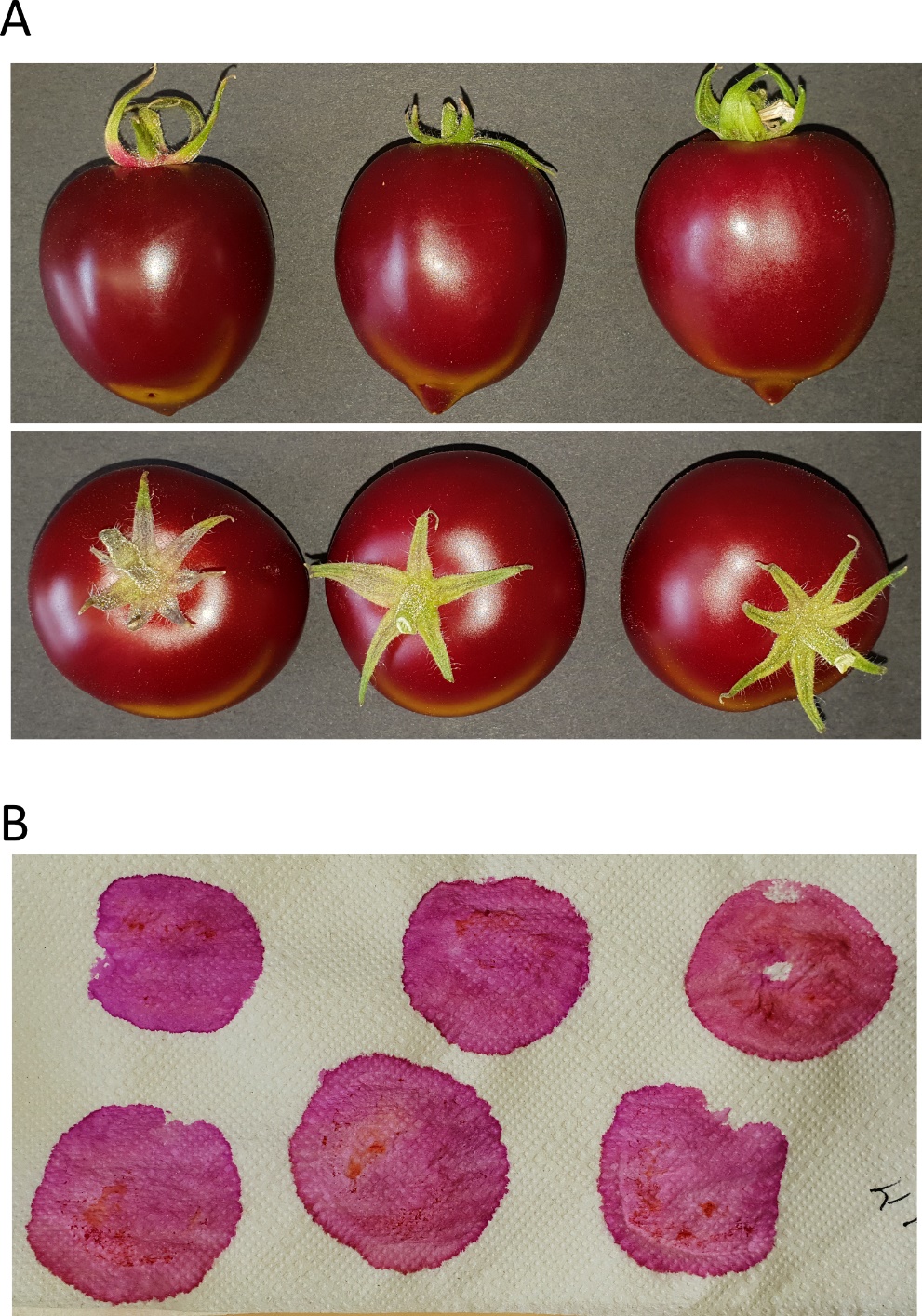
**

**Supplementary Figure 14: Plasmid stability of high copy plasmids in *E.coli* and Agrobacterium.**

Two plasmid clones of the same construct, pAGM51753 and pAGM51751, were transformed in E.coli in parallel. Plasmids from 12 randomly picked colonies for each transformation were analyzed by restriction digest with NheI and SpeI. pAGM51753 and pAGM51751 were also transformed in Agrobacterium. A miniprep made from each transformation was retransformed in E.coli, and 12 randomly picked colonies were analyzed by restriction digest with NheI and SpeI.


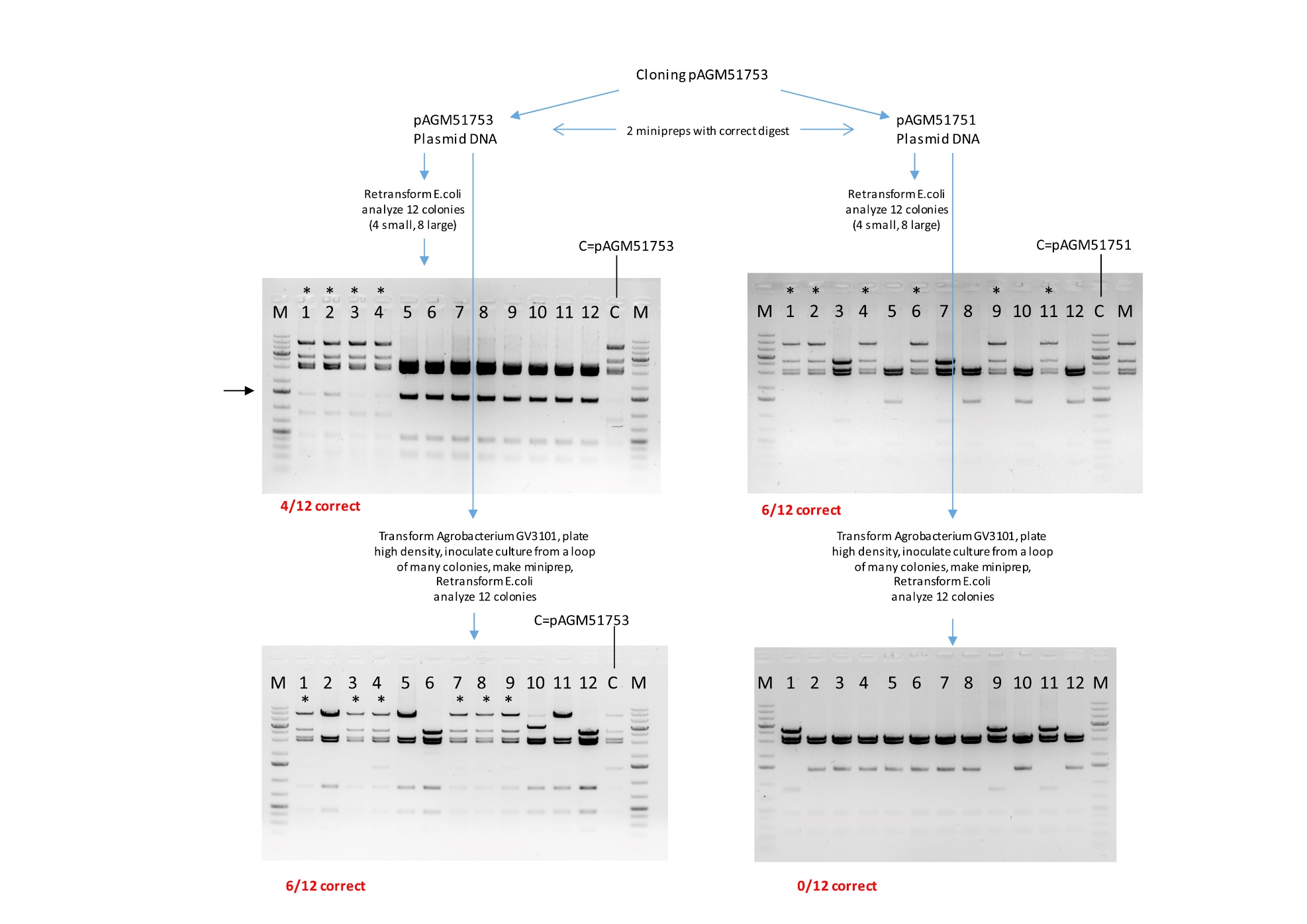


**Supplementary Figure 15: Plasmid stability of low copy plasmids in *E.coli* and Agrobacterium.**

Two plasmid clones of the same construct, pAGM70661 and pAGM70666, were transformed in E.coli in parallel. Plasmids from 12 randomly picked colonies for each transformation were analyzed by restriction digest with NheI and SpeI. pAGM70661 and pAGM70666 were also transformed in Agrobacterium. A miniprep made from each transformation was retransformed in E.coli, and 12 randomly picked colonies were analyzed by restriction digest with NheI and SpeI

**
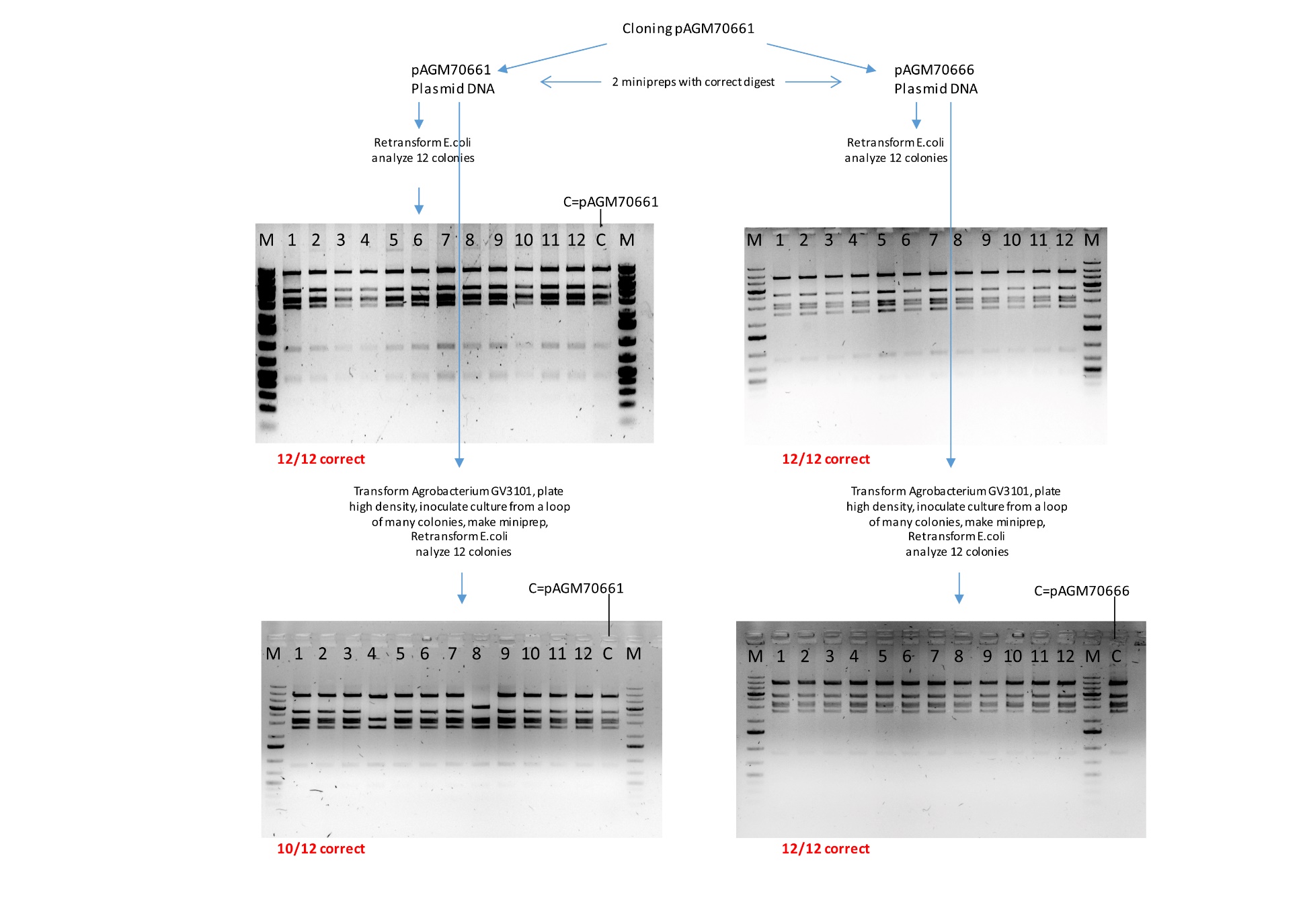
**
